# Identification of Important Effector Proteins in the FOXJ1 Transcriptional Network Associated With Ciliogenesis and Ciliary Function

**DOI:** 10.3389/fgene.2019.00023

**Published:** 2019-03-01

**Authors:** Ishita Mukherjee, Sudipto Roy, Saikat Chakrabarti

**Affiliations:** ^1^Translational Research Unit of Excellence, Structural Biology and Bioinformatics Division, Council for Scientific and Industrial Research – Indian Institute of Chemical Biology, Kolkata, India; ^2^Institute of Molecular and Cell Biology, Singapore, Singapore; ^3^Department of Paediatrics, Yong Loo Lin School of Medicine, National University of Singapore, Singapore, Singapore; ^4^Department of Biological Sciences, National University of Singapore, Singapore, Singapore

**Keywords:** FOXJ1, motile cilia, primary ciliary dyskinesia, ciliopathy, transcriptional network, protein–protein interaction, network analysis

## Abstract

Developmental defects in motile cilia, arising from genetic abnormalities in one or more ciliary genes, can lead to a common ciliopathy known as primary ciliary dyskinesia (PCD). Functional studies in model organisms undertaken to understand PCD or cilia biogenesis have identified 100s of genes regulated by Foxj1, the master regulator of motile ciliogenesis. However, limited systems based studies have been performed to elucidate proteins or network/s crucial to the motile ciliary interactome, although this approach holds promise for identification of multiple cilia-associated genes, which, in turn, could be utilized for screening and early diagnosis of the disease. Here, based on the assumption that FOXJ1-mediated regulatory and signaling networks are representative of the motile cilia interactome, we have constructed and analyzed the gene regulatory and protein–protein interaction network (PPIN) mediated by FOXJ1. The predicted FOXJ1 regulatory network comprises of 424 directly and 148 indirectly regulated genes. Additionally, based on gene ontology analysis, we have associated 17 directly and 6 indirectly regulated genes with possible ciliary roles. Topological and perturbation analyses of the PPIN (6927 proteins, 40,608 interactions) identified 121 proteins expressed in ciliated cells, which interact with multiple proteins encoded by FoxJ1 induced genes (FIG) as important interacting proteins (IIP). However, it is plausible that IIP transcriptionally regulated by FOXJ1 and/or differentially expressed in PCD are likely to have crucial roles in motile cilia. We have found 20 de-regulated topologically important effector proteins in the FOXJ1 regulatory network, among which some (PLSCR1, SSX2IP, ACTN2, CDC42, HSP90AA1, PIAS4) have previously reported ciliary roles. Furthermore, based on pathway enrichment of these proteins and their primary interactors, we have rationalized their possible roles in the ciliary interactome. For instance, 5 among these novel proteins that are involved in cilia associated signaling pathways (like Notch, Wnt, Hedgehog, Toll-like receptor etc.) could be ‘topologically important signaling proteins.’ Therefore, based on this FOXJ1 network study we have predicted important effectors in the motile cilia interactome, which are possibly associated with ciliary biology and/or function and are likely to further our understanding of the pathophysiology in ciliopathies like PCD.

## Introduction

Cilia, microtubule based hair-like organelles, are primarily composed of a structural core, the axoneme, in addition to the basal body, transition zone, ciliary membrane and the ciliary tip (Fliegauf et al., 2007). Macromolecular synthesis and assembly of all of these ciliary structures is a complex and co-ordinately regulated process that involves intraflagellar transport (except cytosolic ciliogenesis), membrane trafficking and selective import of ciliary proteins through the transition zone (Ishikawa and Marshall, 2011). Based on their axonemal organization, 9+2 microtubular architecture with dynein arms or 9+0 without dynein, cilia can be either motile or non-motile, respectively. Both of these kinds of cilia have diverse tissue specific roles in different physiological and developmental processes like cellular motility, fluid clearance, sensory reception, and signaling (Bisgrove and Yost, 2006; Fliegauf et al., 2007). Given their complexity, mutation(s) or defect(s) in one or more proteins involved in the structural organization of cilia or regulation of their assembly can result in abnormalities in the formation or function of these organelles (Horani et al., 2016). These defects in cilia formation or function result in disrupted development of body pattern or physiology of multiple organ systems (Bisgrove and Yost, 2006; Ishikawa and Marshall, 2011) leading to a range of disorders collectively referred to as ‘ciliopathies.’ In particular, this spectrum of disorders could be associated with immotile/primary cilia like polycystic kidney diseases, nephronophthisis, Bardet-Biedl syndrome etc. or with motile cilia like primary ciliary dyskinesia (PCD) (Bisgrove and Yost, 2006).

PCD, the most prevalent of ciliopathies, is a genetically heterogeneous disorder, clinically associated with chronic respiratory infections, bronchiectasis, infertility and in certain cases, hydrocephalus or laterality defects (Zariwala et al., 2011). However, PCD exhibits variability in clinical phenotype, and further, mutations in all disease causing genes may not be exhibited as defects in ciliary ultrastructure. Thus, a genetic screening test for PCD causing genes could be helpful for disease diagnosis (Zariwala et al., 2011). In this respect, the genetic basis of PCD, which is usually inherited as an autosomal recessive trait, has been studied with the help of conventional family based, genome-wide linkage studies, candidate gene testing, homozygosity mapping as well as genome and exome sequencing studies to identify causative mutations (Zariwala et al., 2011; Horani et al., 2016). In addition, while identification of PCD causing genes with conventional studies (family based or genome-wide linkage analysis) has been challenging due to locus heterogeneity, nevertheless, different sequencing approaches have identified multiple disease causing genes in families of PCD patients during the last decade (Zariwala et al., 2011; Horani et al., 2016). At present, the OMIM database lists about 35 disease causing genes with mutations associated with PCD (McKusick, 1998; Amberger et al., 2015).

However, such disease causing variants identified with the help of sequencing could be family specific (Horani et al., 2016), and moreover, such approaches may only be useful to study certain cases that have been successfully diagnosed. Thus, other complementary approaches in model organisms, which explore cilia biogenesis to identify genes or proteins important in cilia formation or function, have also been undertaken (for example, May-Simera et al., 2016; Rao et al., 2016; Terré et al., 2016). In addition, some large scale studies have identified thousands of proteins in the ciliary proteome that co-ordinately interact to form these organelles (Ishikawa et al., 2012; Boldt et al., 2016), and such cascades of interactions are regulated by transcription factors like GemC1, McIdas, E2f4, E2f5, Myb, Rfx1, Rfx2, Rfx3, Rfx4, and FoxJ1 (Choksi et al., 2014b; Arbi et al., 2016; Danielian et al., 2016; Vladar and Mitchell, 2016). Further, while Rfx factors regulate both motile and immotile cilia genes, FoxJ1 specifically regulates motile cilia biogenesis, and appears to be its master regulator (Choksi et al., 2014b). This is because FoxJ1 regulates a set of genes known as FoxJ1 induced genes (FIG), which together are sufficient for motile cilia development and function (Stubbs et al., 2008; Yu et al., 2008; Choksi et al., 2014a).

In this study, our primary objective lay in studying the motile cilia interactome to identify possible essential proteins and their probable functions in this interactome. In this respect, we have studied two components of the motile cilia interactome, a probable transcriptional network and a probable signaling network. The transcriptional network in the motile cilia interactome that we have considered here is the FOXJ1 regulatory network. For this purpose, we have predicted the regulatory network of the motile cilia master regulator, FOXJ1 and annotated the network genes based on information from different ciliary reference databases. Based on this analysis, however, we found that while ∼83% of the regulatory network genes are expressed in multiple motile ciliated tissues, only ∼24% of them are presently annotated. Further, the annotated network genes mainly comprised of ciliary structural component proteins or motility associated proteins. As mentioned above, it has been established in previous studies that FoxJ1 over-expression is sufficient to drive the motile ciliogenic program and generate functional ectopic motile cilia (Stubbs et al., 2008; Yu et al., 2008; Choksi et al., 2014a). It is possible that the FIG encoded protein (FIGp) participate in motile cilia assembly or function in a co-ordinated manner in association with other proteins (signaling) of the ciliary milieu. In this context, we next sought to study the representative motile cilia interactome comprised of the regulatory network proteins and their protein–protein interaction network (PPIN) with different graph theory metrics. This analysis was performed in order to identify the key connector proteins (regulatory network proteins) that relay the information onto the signaling component/s during motile cilia biogenesis. Further, the topological analysis has been complemented with a functional analysis to determine whether these proteins could indeed be essential for ciliogenesis or ciliary function. Traditionally, such essential proteins have been identified with the help of gene misexpression, targeted gene knock-out or knock-down studies in experimental model systems (Stubbs et al., 2008; Yu et al., 2008; Choksi et al., 2014a; May-Simera et al., 2016; Terré et al., 2016). By contrast, in this study we have utilized an *in silico* knock-out strategy, and determined the effects on the motile cilia interactome by deriving whether the effective change in a centrality measure as a result of the knock-out varied significantly. Moreover, in order to ascertain the relevance of these predicted essential proteins to ciliary biology, we have utilized literature-based evidences to determine whether some of the proteins have previously identified involvements in ciliary biology. Finally, to determine the likely functions of these proteins, we have utilized the concept of ‘guilt by association’ (which states that two proteins that are known to interact with one another, may usually participate in the same or similar cellular functions; Oliver, 2000; Schwikowski et al., 2000), and determined the enriched pathways or processes among the proteins of interest.

Thus, studying the PPIN associated with the FOXJ1 regulatory network might help us in elucidating the topologically important effector proteins that lie at the interface of the FOXJ1 regulatory network and the associated protein interaction network. These proteins might form a crucial link between the FOXJ1 regulatory and cilia biogenesis-associated signaling components in the motile cilium, and mediate some of the functions of FOXJ1 and its regulatory network. Importantly, such proteins identified in this manner could be crucial for ciliary development or maintenance of ciliary function, and one could screen for defects in this repertoire of proteins to determine possible causal or etiological genes for PCD.

## Materials and Methods

### Collating an Information Resource Regarding Cilia Biogenesis

Genes experimentally probed and identified to be involved in ciliogenesis or ciliary function were collected from different studies and databases like the SysCilia gold standard database (van Dam et al., 2013), Reactome pathway database [R-HSA-5617833] (Croft et al., 2014; Fabregat et al., 2018), FIG study (Choksi et al., 2014a), cilliary proteome related studies (Gupta et al., 2015; Boldt et al., 2016) and OMIM database (McKusick, 1998; Amberger et al., 2015). This resource has been subsequently utilized to summarize the previously identified involvement(s) of the FOXJ1 transcriptional network genes. It has also been utilized as a preliminary validation resource to ascertain whether certain genes predicted to be involved in ciliogenesis or ciliary function by our computational approach are actually involved in the process.

### Cilia Associated Expression Analysis

In order to prepare a set of disease (PCD) associated genes, we have considered a dataset available from a previous study that explored the expression profile of bronchial tissue of PCD patients (Geremek et al., 2014). Differentially expressed genes were determined with the help of limma (Ritchie et al., 2015) R package in Gene Expression Omnibus (GEO) series dataset (GSE25186) (Edgar et al., 2002; Barrett et al., 2013; Geremek et al., 2014). Genes having fold change ≥ 2 and *p*-value ≤ 0.05 were considered as differentially expressed and possibly associated with PCD based on the considered PCD case study. Databases or datasets [e.g., Choksi et al. (2014a) expression study, CilDB (Arnaiz et al., 2009; Arnaiz et al., 2014), PCD expression analysis case study (Geremek et al., 2014) and Human Protein Atlas (Uhlen et al., 2010; Uhlén et al., 2015; Thul et al., 2017)] providing evidence for RNA or protein expression abundance in ciliary cells were taken into consideration for ‘cilia associated expression analysis.’ For this, if genes had expression information in the ‘cilia associated expression analysis,’ they were considered to have possible associations with ciliary biology.

### Constructing the FOXJ1 Regulatory Network

Transcription factor binding sites may generally be predicted by scanning a position weight matrix (PWM) against DNA using a pattern matching algorithm (Bulyk, 2004). Genes which are likely to be regulated transcriptionally by FOXJ1 were predicted with the help of Rsat (Turatsinze et al., 2008). An initial set of genes (FIG) to be studied was prepared based on their induction upon FoxJ1 over-expression in the zebrafish (Choksi et al., 2014a). With the help of the Ensembl Compara database we could determine that these FIG have high confidence orthologs in humans (*Homo sapiens*) and mice (*Mus musculus*) (Herrero et al., 2016). Further, for prediction of transcription factor binding motifs, pre-requisites include a PWM for the transcription factor and a background matrix representative of general base frequencies around the transcription start site (TSS) of genes. It is possible that orthologous transcription factors from human and mouse may share similar binding specificities (Jolma et al., 2013). Thus, a PWM for FoxJ1 (mouse) [PB0016.1] was collected from footprintDB database (Sebastian and Contreras-Moreira, 2014) since PWM for human FOXJ1 is unavailable. It was observed that these proteins are 92.6% identical, and moreover, the DNA binding domains are 100% identical (Supplementary Figure 1A), which further suggested that these proteins may share similar binding specificities. Further, a background model (Markov order), representative of ±6 kb of random *Homo sapiens* genes, was prepared. These were then utilized to scan ±6 kb of the FIG for the presence of FOXJ1 binding motif (Medina-Rivera et al., 2015). Predicted binding sites having *p*-value ≤ 1e^−04^ were considered to be genes directly regulated by FOXJ1. Further, a logoplot (R Core Team, 2016) representative of the binding specificity of FOXJ1 (human) was prepared from the multiple sequence alignment of the predicted FOXJ1 binding sites among human orthologs of FIG.

### Determining Ciliary Functional Associations of FOXJ1 Regulatory Network Genes Based on Gene Ontology (GO) Analysis

Based on the CCR dataset we could ascertain the ciliary roles of some of the FOXJ1 regulatoy network genes. However, we further performed GO analysis and GO enrichment analysis in order to assign probable functional relevance to the remaining genes. GO analysis was performed using DAVID web server (Huang da et al., 2009b), and with the help of FGNet (Aibar et al., 2015), certain GO based enriched clusters among the genes were determined. Functions of genes belonging to clusters having *p*-value ≤ 1e^−02^, cluster enrichment score ≥ 2, fold enrichment ≥ 4 could be predicted based on this analysis.

### Constructing the FOXJ1 Associated Ciliary Interactome

In order to prepare a PPIN representative of proteins and connections important for cilia structure or function in relation to FOXJ1 activation, we considered the FIGp as seed proteins. In particular, a PPIN is comprised of proteins as nodes, and two proteins are connected by an edge if they are known to be interacting. Thus, a PPIN was constructed around these seed proteins by obtaining high confidence experimentally reported interactions between these proteins and other proteins from SysCilia (van Dam et al., 2013), Bioplex (Huttlin et al., 2015), STRING (Szklarczyk et al., 2015), and BioGrid (Stark et al., 2006; Chatr-aryamontri et al., 2017) databases. In this way, a network of primary interactors of FIGp was constructed, and the largest connected component of this network was extracted (FIG-sub-network). We then analyzed the degree distribution of the FIG-sub-network to determine whether the constructed network was a scale free network wherein the degree distribution follows a power law. The degree (k: number of proteins each protein is connected to) of each protein in the network was computed and a power law [*P(k)∼ k^−α^* where α is the degree exponent] was fitted to the resulting distribution. A Kolmogorov–Smirnov test (which computes a *p*-value for the estimated power-law fit to the data) was used to determine the goodness of fit of the degree distributions to the power law (at 0.1 level of significance) (Clauset et al., 2009; R Core Team, 2016).

### Identifying Topologically Important or Essential Proteins in the Representative Motile Cilia Interactome (FIG-sub-network)

Once we had a PPIN representative of motile cilia interactome in hand, we analyzed the FIG-sub-network based on a computationally faster implementation of a previously proposed methodology (Bhattacharyya and Chakrabarti, 2015). With the help of this analysis we have identified topologically important proteins in this network. For this purpose, we have considered different graph theory metrics like degree, shortest path and centrality to determine important interacting proteins (IIP) (combination of hub, bottleneck, central, local network perturbing, and global network perturbing proteins) in our FIG-sub-network as outlined below.

#### Node Perturbation Analysis of the FIG-sub-network

Previously, it has been found that removal of hub proteins has a significant effect on the topology of the PPIN, while they are extremely resilient toward the removal of random nodes (Barabási and Oltvai, 2004). Based on this observation, we have previously devised a centrality measure which tries to capture the change in the topology of the network on *in silico* node removal to identify topologically important proteins in a protein interaction network (Bhattacharyya and Chakrabarti, 2015). Thus, with the objective of identifying topologically important proteins, a node perturbation analysis of the global network and local sub-graphs in the FIG-sub-network was performed. The local sub-graphs comprised of proteins having degree higher than 2, and their 2nd level interactors and the local network centrality measures of the nodes before and after node removal in the local sub-graph were compared. It was assumed that higher the difference in the scores (LNCS), higher is the perturbation ability, and thus, proteins important for maintaining the integrity of the local sub-network determined in this manner were termed as local network perturbing proteins (LNPP). Similarly, global network perturbation was performed by removing a single node at a time and studying its effect on the global network centrality score (GNCS) before and after the perturbation. Proteins identified as crucial for maintaining the global sub-network integrity, based on the difference in the GNCS scores before and after perturbation, were termed as global network perturbing proteins (GNPP).

$$\begin{array}{c} \mathrm{CS}=\sum C(\mathrm{betweeness})+C(\mathrm{closeness})+C(\mathrm{clustering coeficient})\\ \mathrm{CCS}=\sum_{1}^{n}\mathrm{CS}\\ \mathrm{LNCS}=1/N\sum_{1}^{N}\mathrm{CCS} \end{array}$$

where *n* is the number of first degree interactors, *CS* is the combined score, *CCS* is the cumulative centrality score, *LNCS* is the local network centrality score and *N* is the number of nodes in local sub graph. The LNCS scores were normalized into z-score and nodes having *z*-score ≥1 were considered as LNPP.

$$\mathrm{GNCS}=1/N\sum_{1}^{N}\mathrm{CCS}$$

where *GNCS* is the global network centrality score and N is the number of nodes in the global network. The GNCS scores were normalized into *z*-score and nodes having *z*-score ≥ 0.5 were considered as GNPP.

#### Identification of Hub and Bottleneck Proteins

Analyses of different biological PPINs have identified that hub and bottleneck proteins, which are determined by graph theory calculations that measure inherent properties of scale free networks, could indeed be essential proteins (Barabási and Oltvai, 2004; Albert, 2005; Yu et al., 2007). For calculating hubs, the node degrees were normalized into *z*-scores and the fraction of degree population having *z*-score ≥2 was considered as having significantly higher degree than the rest of the population, and protein nodes having degree 57 or higher were considered as hub proteins. Additionally, bottleneck proteins which have a high betweenness centrality value (multiple “shortest paths” passing through them) could be key connector proteins (Yu et al., 2007). Herein, proteins having betweenness centrality indices higher than two standard deviations from the mean of the betweenness centrality distribution were considered as bottleneck proteins.

#### Centrality Analysis of the FIG-sub-network

Compactness of a network and capability of relaying information can be further assessed with the help of another graph theory based concept, for instance, centrality (Pavlopoulos et al., 2011). It is possible to identify proteins which could be of biological significance with the help of centrality analysis, since previous reports have suggested that the removal of central proteins by gene deletion may lead to lethal phenotypic consequences (Jeong et al., 2001). Thus herein, we have considered a range of centrality indices to identify central proteins with possible biological significance in our FIG-sub-network. Centrality indices like closeness, load, eigen centrality and clustering coefficient were evaluated and combined to derive an average centrality parameter (combined score, CS) for each node. The CS and the cumulative centrality score (CCS) were computed as shown below:

$$\begin{array}{lll} \mathrm{CS} & = & \sum C(\mathrm{load})+C(\mathrm{closeness})+C(\mathrm{eigen vector})\\ & & +C(\mathrm{clustering coeficient})\\ \mathrm{CCS} & = & \sum_{1}^{n}\mathrm{CS} \end{array}$$

where *n* is the number of first degree interactors, *CS* is the combined score and *CCS* is the cumulative centrality score. The CCS scores were normalized into *z*-score and nodes having *z*-score ≥ 2 were considered as central proteins.

#### IIP in the FIG-sub-network

Based on the assumption that proteins identified as topologically important in two or more categories (hub, bottleneck, central, local network perturbing, and global network perturbing) could be essential proteins in the FIG-sub-network, we have categorized them as IIP. Only proteins with expression information support in ciliated cells based on ‘cilia associated expression analysis’ were retained as IIP. Additionally, any overlap between the IIP and the CCR dataset may be suggestive of their functional relevance in the ciliary milieu. Moreover, in order to ascertain the probable functional role(s) of these IIP in association with FIGp, we have estimated the enriched pathways among interacting FIGp, IIP and their direct interactors with the help of an R package (Yu and He, 2016).

### Identifying Important Effector Proteins by Comparing FOXJ1 Associated Transcriptional and PPINs

IIP which could be associated with ciliogenesis or PCD based on their differential expression status upon ectopic FoxJ1 expression in zebrafish or in PCD were identified as important effectors in FOXJ1 regulatory network. Further, these topologically important effector proteins are likely to be involved in a range of cellular pathways particularly signaling pathways. Pathway enrichment analysis with *p*-value cut off of 1e^−06^ was performed in ReactomePA (Yu and He, 2016) considering the effectors and their primary interactors. Proteins participating in enriched ‘cilia associated signaling pathways’ were predicted to have possible ciliary association. In this analysis, we have considered ‘cilia associated signaling pathways’ such as cell cycle (Quarmby and Parker, 2005; Izawa et al., 2015), TGF-beta (Clement et al., 2013), FGF (Neugebauer et al., 2009), RHO GTPase (Kim et al., 2015), Hedgehog, PDGF, WNT (Goetz and Anderson, 2010), TLR signaling (Baek et al., 2017) and vesicle mediated transport (Nachury et al., 2010), since all of these are known to have an association with cilia. The pathway enrichment analysis was complemented with GO mapping in DAVID (Huang da et al., 2009b). Proteins associated with GO categories associated with cilia biology like cilium morphogenesis, cell cycle (Quarmby and Parker, 2005; Izawa et al., 2015), actin organization (cytoskeleton organization, actin filament organization), protein ubiquitination, centrosome cycle, protein folding (heat shock proteins) and establishment or maintenance of cell polarity (Stephens and Lemieux, 1999; Pan et al., 2007; Nachury et al., 2010; Bettencourt-Dias et al., 2011; Jones et al., 2012; Prodromou et al., 2012; Kasahara et al., 2014; May-Simera et al., 2016; Shearer and Saunders, 2016; Kohli et al., 2017) were predicted to have possible ciliary association.

### Determining the Relevance of the Predicted IIP in the Ciliary Interactome to Ciliary Biology

Literature based evidences of the involvements of the IIP in ciliary biology or gene expression based association of the IIP with PCD were considered as preliminary supportive evidences toward the relevance of the computational predictions to ciliary biology. In order to determine the significance of the finding that some of the IIP were found to be differentially expressed in PCD patients, we have performed a randomization analysis. In each trial, a 121 proteins were randomly selected from the set of proteins in FIG-sub-network and matched to the set of differentially expressed PCD proteins in our network. Based on the number of matches obtained in a 1000 trials, a *z*-test was performed to determine whether the association between the IIP and PCD expression status that we had observed was significant. Additionally, a few of the IIP proteins have previously reported ciliary roles in literature. A similar randomization analysis was performed considering matches with the CCR and the significance of this association was also determined.

## Results

### FOXJ1 Regulatory Network Governing Motile Cilia Biogenesis and Function

Functional genomics studies have identified the FoxJ1 protein as the master regulator of motile ciliogenesis, and it is crucial for ciliary axoneme assembly, basal body docking and ciliary motility (Stubbs et al., 2008; Yu et al., 2008; Choksi et al., 2014a). Over-expression of FoxJ1 in model systems such as the zebrafish and *Xenopus* appears to be necessary and sufficient for the development of motile cilia (Stubbs et al., 2008; Yu et al., 2008). Therefore, determining the predicted regulatory network of FOXJ1, might in turn, help us to better understand ciliogenesis and ciliopathies associated with abnormal ciliary differentiation and function in humans. In order to study the regulatory network that is essential in motile cilia development, we have predicted the genes that are likely to be transcriptionally regulated, directly or indirectly, by FOXJ1 and associated them with known or probable ciliary roles. Based on the presence of FOXJ1 *cis*-regulatory sites in upstream/downstream region of transcription start sites of FIG (Choksi et al., 2014a), we could identify that a large fraction of the FIG (424/572) are directly regulated by FOXJ1 (Supplementary Figure 1B and Supplementary Table 1). It is likely that the other 148 induced genes either contain FOXJ1 binding sites in distant enhancers or are indirectly regulated by FOXJ1 via other transcription factors directly activated by FOXJ1. Further, the FOXJ1 protein appears to have a binding preference toward the consensus sequence NNN[GA]TAAACAAANNNN (Supplementary Figure 1C). Additionally, functional annotations for these genes were retrieved from the CCR, and the identified known ciliary associations for genes directly and indirectly regulated by FOXJ1 were classified into functional cohorts (Assigned Ciliary Role) manually (Supplementary Table 2).

#### Additional Ciliary Association for FOXJ1 Regulatory Network Genes Based on GO Analysis

Functional enrichment analysis of proteins associating with one another under a particular context may provide an idea regarding the probable collective activities and the most likely functions that these proteins may have in this context. At the outset, GO mapping elucidated 20 transcription factors (associated with DNA binding or transcription factor ontology class) among the FIGp (Supplementary Table 2). Further, predicted ciliary associations were determined based on GO enrichment analysis (Supplementary Table 3). In the GO mapped data, we found groups of genes having similar functions (co-associated genes) (Huang da et al., 2009a,b) belonging to multiple GO annotation categories, and such co-associated genes from different annotation clusters were grouped and categorized into common ‘GO based Ciliary Association(s)’ (Supplementary Table 3).

**FIGURE 1:**
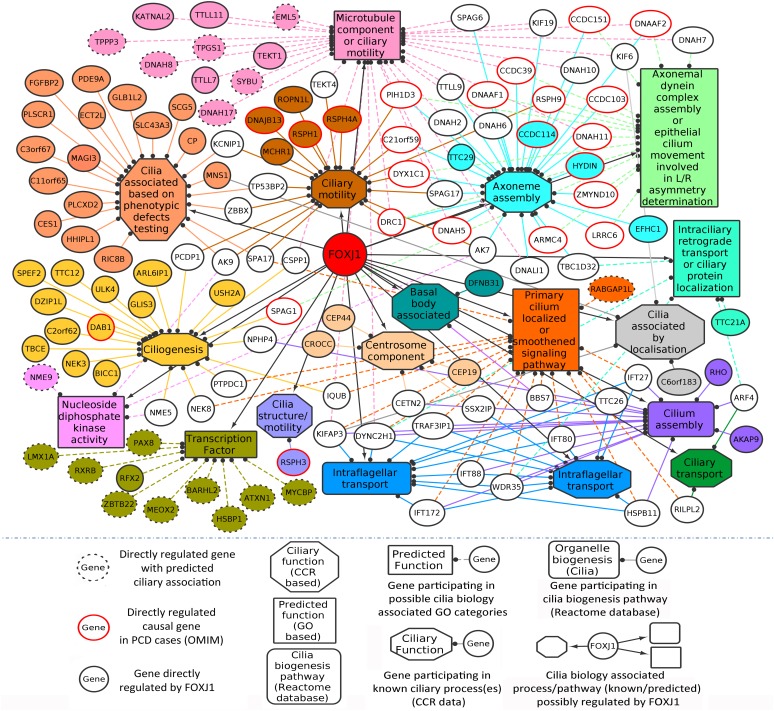
Directly regulated genes in the predicted FOXJ1 gene regulatory network and possible ciliary processes mediated by them. FOXJ1 directly regulates genes that are involved in ciliary structure assembly and motility, as depicted here. Known ciliary associations were derived from the collated ciliary resource. Additionally, the probable roles of some genes predicted based on gene ontology analysis have also been included here. The genes and edges are color coded to indicate the processes the genes regulate or are involved in (genes associated with more than one process are not colored).

In this manner, we were able to assign possible ciliary roles to 102 (directly) and 35 (indirectly) regulated genes based on the CCR dataset (Supplementary Table 2), and 17 (directly) and 6 (indirectly) regulated genes with the help of the GO analysis (Table 1). Based on the CCR dataset based annotation, we could assign ciliary associations such as participating in ‘ciliary structural assembly or motility’ for most of the directly (82) and indirectly (26) regulated FOXJ1 target genes (Figure 1 and Supplementary Figure 2). Briefly, this analysis elucidated that FOXJ1 primarily influences ‘ciliary structural assembly or motility’ by regulating three classes of proteins. These classes include ‘proteins that act as structural constituents of cilia’ (axoneme assembly, IFT complex, centrosome component, basal body associated), ‘proteins that regulate the structural assembly of cilia’ (cilia assembly, ciliogenesis) and ‘proteins that have a role in ciliary function’ (like ciliary transport or motility) (Figure 1, Supplementary Figure 2, and Supplementary Table 2). A fraction of directly regulated (24.06%) and indirectly regulated (23.65%) FIG shared an overlap with the CCR dataset, and as such many FIG could not be associated with ciliary roles in this manner (Supplementary Table 2). Thus, having studied the FOXJ1 transcriptional network, it became apparent that the majority of genes that have been identified or extensively characterized are structural components of cilia. However, based on ‘cilia associated expression analysis,’ 84.67% of directly and 81.76% of indirectly regulated genes were found to be expressed in multiple motile ciliated tissues and some are differentially expressed in PCD (Supplementary Figures 3A–C and Supplementary Table 2). Further, it has been established that signaling pathways like Notch, Fgf and Wnt (Neugebauer et al., 2009; Lopes et al., 2010; Caron et al., 2012) are known to be involved in motile cilia biology. In this context, we were interested in studying the regulatory network proteins in a broader context including the associated protein–protein interactions, in order to identify the key connector proteins (regulatory network proteins) that relay the information onto the signaling component within the cell. Additionally, it is possible that disruptions in some of these network interactions or genes causing PCD may alter the motile cilia interactome, in turn leading to ciliopathies. Therefore, we were interested in studying the probable signaling network/s acting concurrently or in response to FOXJ1 activation involved in this process. In order to achieve this, we have subsequently analyzed the PPIN associated with FOXJ1 and its induced proteins, and studied the probable role(s) of the identified essential or effector proteins in the network.

**Table 1:** Novel predicted functions of FOXJ1 regulated genes based on gene ontology analysis.

S. No	FOXJ1 Target Gene	^1^Effect	^2^Assigned Ciliary Role
1	RABGAP1L	Direct	Cilia associated by localization
2	DNAH8	Direct	Ciliary structure/motility
3	TPPP3	Direct	Ciliary structure/motility
4	NME9	Direct	Ciliary structure/motility
5	DNAH17	Direct	Ciliary structure/motility
6	TPGS1	Direct	Ciliary structure/motility
7	EML5	Direct	Ciliary structure/motility
8	SYBU	Direct	Ciliary structure/motility
9	HSBP1	Direct	Regulates genes involved in ciliary assembly/motility (Transcription factor)
10	MYCBP	Direct	Regulates genes involved in ciliary assembly/motility (Transcription factor)
11	ATXN1	Direct	Regulates genes involved in ciliary assembly/motility (Transcription factor)
12	BARHL2	Direct	Regulates genes involved in ciliary assembly/motility (Transcription factor)
13	LMX1A	Direct	Regulates genes involved in ciliary assembly/motility (Transcription factor)
14	MEOX2	Direct	Regulates genes involved in ciliary assembly/motility (Transcription factor)
15	PAX8	Direct	Regulates genes involved in ciliary assembly/motility (Transcription factor)
16	RXRB	Direct	Regulates genes involved in ciliary assembly/motility (Transcription factor)
17	ZBTB22	Direct	Regulates genes involved in ciliary assembly/motility (Transcription factor)
18	TUBA3D	Indirect	Ciliary structure/motility
19	NR1H2	Indirect	Regulates genes involved in ciliary assembly/motility (Transcription factor)
20	FOSB	Indirect	Regulates genes involved in ciliary assembly/motility (Transcription factor)
21	ATF5	Indirect	Regulates genes involved in ciliary assembly/motility (Transcription factor)
22	CHD4	Indirect	Regulates genes involved in ciliary assembly/motility (Transcription factor)
23	ELF3	Indirect	Regulates genes involved in ciliary assembly/motility (Transcription factor)

*^1^Effect: Direct/Indirect depending on whether the gene is directly or indirectly regulated by FOXJ1. ^2^Assigned Ciliary Role: Predicted ciliary role based on gene ontology analysis.*

### Essential or Important Interacting Proteins in Representative Motile Cilia Network

**FIGURE 2:**
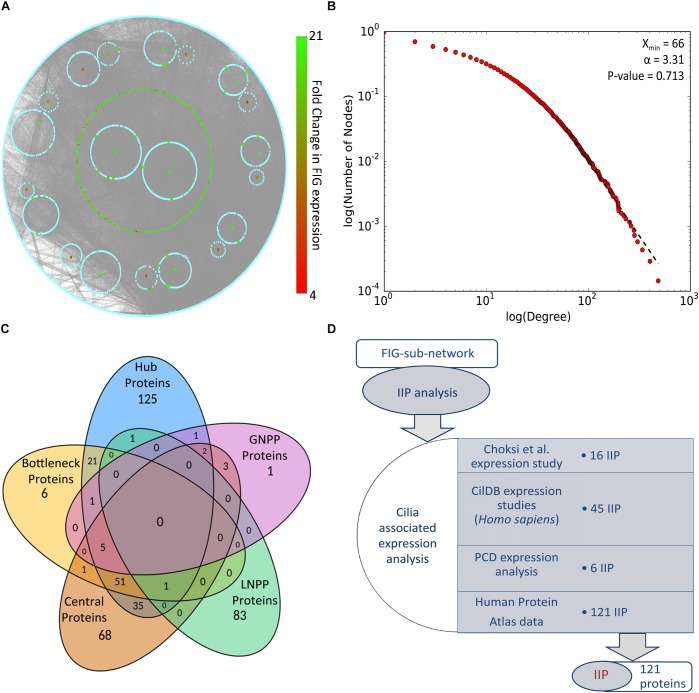
PPIN analysis of primary interaction network of FIG-sub-network proteins. **(A)** The scale free network comprising of largest connected component of primary interaction network of FIGp considered for the IIP analysis is shown here. **(B)** The degree distribution plot and the p-value for goodness of fit of the power law to the degree distribution as determined by the Kolmogorov-Smirnov test is represented here. **(C)** The number of proteins identified in each category (hub, bottleneck, central, local network perturbing and global network perturbing protein) considered during IIP analysis are indicated in the Venn diagram. **(D)** The number of proteins that could be associated with cilia based on ‘cilia associated expression analysis’ that were considered as IIP is shown here. Abbreviations: FIG, FoxJ1 induced genes; FIGp, FIG encoded protein; IIP, Important interacting proteins; PPIN, protein-protein interaction network.

Identification of IIP in the FOXJ1 regulatory network or possible essential proteins in the motile cilia protein interactome may be achieved with extensive computational analysis of the PPIN likely to be associated with the FIGp. Scale free biological networks that follow a power law exhibit certain characteristic topological properties, which may be studied with different graph theory based metrics in pursuance of inferences regarding the PPIN (Barabási and Oltvai, 2004; Yook et al., 2004; Yu et al., 2007). Therefore, with the help of high confidence physical interaction data from different protein-protein interaction databases including cilia specific datasets, a re-constructed PPIN (FIG-sub-network) was prepared. The FIG-sub-network comprised of 6493 primary interactors of FIGp (434) and their first level interactors participating in 40,608 interactions (Figure 2A). Networks conforming to the power law must have *p*-value higher than 0.1 (Clauset et al., 2009), and the *p*-value of the estimated fit of the degree distribution to the power law determined herein was found to be 0.71. Thus, it was concluded that the re-constructed network was a scale free network (Figure 2B).

#### Network Analysis of Representative Motile Cilia Interactome (Primary Interaction Network of FIGp)

Topologically important proteins in a scale free PPIN like hub, bottleneck and central proteins, may be identified with the help of different graph theory based measures, and such proteins could be essential for the network integrity or function (Barabási and Oltvai, 2004; Yu et al., 2007; Pavlopoulos et al., 2011). In this respect, *in silico* node deletion that resulted in significant changes in network topology were studied as a measure of centrality, and 85 local network perturbing and 13 global network perturbing proteins were identified (Figure 2C). The overlap among these network perturbing proteins and other topologically important proteins [hub (243), bottleneck (86), and central (166)] was studied, and proteins identified as important in two or more metrics, were identified as IIP (122) (Figure 2C and Supplementary Table 4). Genes may be associated with cilia based on their expression, and such expression-based evidences from multiple studies might further strengthen our assumption that IIP possibly interact with FIGp in the ciliary interactome. We have taken into consideration expression information at the mRNA or protein levels in multiple motile ciliated tissues or differential expression (mRNA) information from studies exploring cilia biogenesis or ciliopathies as indicated in the ‘cilia associated expression analysis’ (Figure 2D). Further, the distribution pattern of the 121 cilia expressed IIP in multiple motile ciliated tissues elucidated that 114 among them were expressed in all ciliated tissues considered here (Supplementary Figure 3D and Supplementary Table 4). Moreover, among these proteins, 6 were found to be associated with PCD based on the differential expression analysis (Supplementary Figure 3E) [the randomization analysis indicated that this observation is significant at 10% level of significance]. Further, the observation that 33 IIP had established roles in ciliogenesis and/or cilia function suggested that the identified IIP could indeed have essential roles in motile cilia or PCD pathogenesis (Supplementary Table 4) [based on the randomization analysis this observation is significant at 1% level of significance].

#### IIP and Their Probable Essential Roles in Motile Cilia Interactome

**FIGURE 3:**
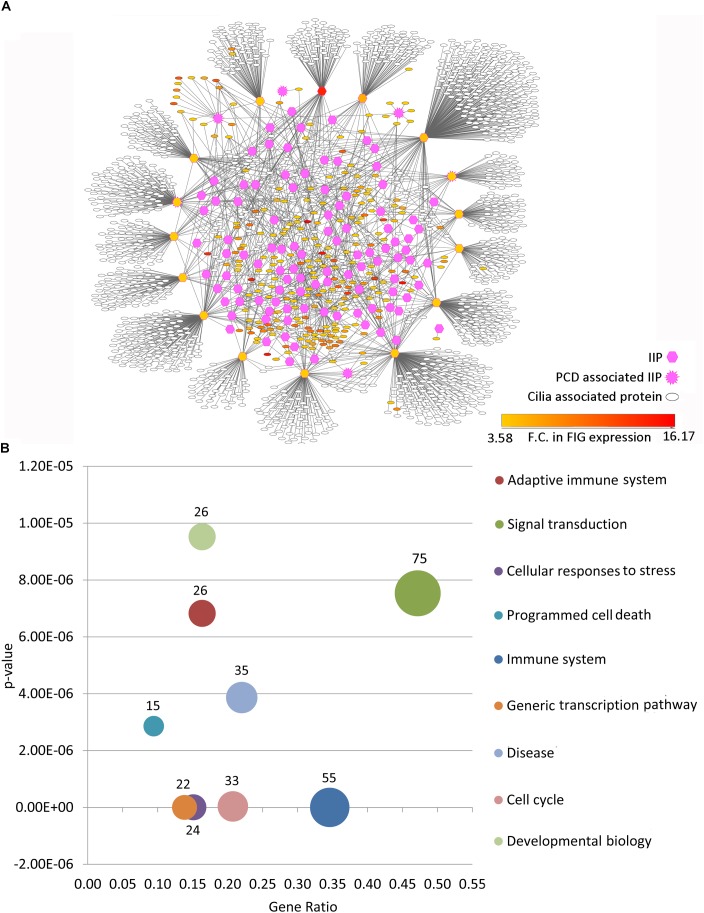
Inter-relationship among IIP and FOXJ1 regulatory network proteins or FIGp. **(A)** The IIP (120) and their primary interactors form an inter-connected module with FIGp (246) within the motile cilia interactome as depicted here. **(B)** Cellular pathways that the inter-connected FOXJ1 regulatory network proteins and IIP (enriched pathways with p-value lower than 1e^−05^) are likely to be involved in are shown. Abbreviations: F.C., Fold change; FIG, FoxJ1 induced genes; FIGp, FIG encoded protein; IIP, Important interacting proteins; PCD, Primary ciliary dyskinesia.

Among the thousands of interacting proteins in the motile cilia interactome, 121 crucial interacting proteins were identified in the representative motile cilia interactome (primary interaction network of FIGp). These IIP form an inter-connected module in the ciliary interactome including 2060 interactions among FIGp (246), IIP (120) and their primary interactors (1666 motile cilia expressed proteins) (Figure 3A). Such IIP that have extensive interactions with FIGp could possibly be involved in the coordinated assembly of functional motile cilia in association with FIGp. Such interacting proteins namely FIGp and IIP, based on the concept of ‘guilt by association’ (Oliver, 2000; Schwikowski et al., 2000), may participate in the same or similar cellular pathways, possibly involved in ciliogenesis. In order to further determine the cellular pathways that the IIP might participate in together with FIGp, we have performed Reactome pathway enrichment analysis among the IIP and FIGp that were found to be interacting. Pathways such as signal transduction, developmental biology, cell cycle, generic transcription pathway, immune system etc. were found to be significantly enriched among these proteins (Figure 3B and Supplementary Table 5). This suggests that in addition to acting as structural components of the ciliary organelle, FIGp, in association with IIP, may participate in different signaling pathways, cell cycle and developmental biology associated processes or regulate transcription of other genes during motile cilia development.

### Important Effector Proteins in the FOXJ1 Regulatory Network Possibly Involved in Ciliary Biology

As outlined above, by extensively analyzing the probable motile cilia interactome, we have determined topologically important proteins in the network. Further, we could identify a module comprised of 246 FIGp and topologically important proteins that are mainly signaling proteins. While such IIP may be essential in the ciliary milieu and possibly functionally relevant, another pertinent question is which proteins in the FOXJ1 regulatory network might act as essential modulators that relay the information onto the signaling component. To address this question, firstly we have considered whether genes that are induced upon FoxJ1 over-expression have been identified as IIP. In this respect, we have found that in particular 16 IIP in the regulatory network interact with multiple other FIG (26), IIP (68) and other expression associated ciliary interactome proteins (1255) (Figure 3A). These FIGp that share extensive interactions with topologically important proteins in the motile cilia interactome are possibly key mediators acting downstream of FOXJ1 activation that in turn participate in ciliogenesis or maintenance of ciliary function. Moreover, genes may be associated with ciliogenesis or PCD based on their differential expression in respiratory epithelial cells of patients with PCD. Interestingly, an additional set of 4 IIP that were found to be differentially expressed in a PCD case study also had associations with multiple FIGp (Figure 3A). Thus, such IIP found to be directly involved in the FOXJ1 regulatory network have been classified as important interacting protein effector (IIP-effector) in the FOXJ1 regulatory network. In addition, IIP that have possible associations with PCD and the FOXJ1 regulatory network (via intermediate FIGp), have also been classified as IIP-effector in the FOXJ1 regulatory network (Table 2). While some of these effector proteins (HSP90AA1, CDC42, ACTN2, SSX2IP, PLSCR1, PIAS4) have previously documented roles in ciliogenesis (Choi et al., 2013; Choksi et al., 2014a; Croft et al., 2014; Klinger et al., 2014; Ramachandran et al., 2015; Kohli et al., 2017; Fabregat et al., 2018), we report here a set of 14 novel proteins that may act as crucial mediators in the FOXJ1 regulatory network (Table 2).

**Table 2:** IIP-effector proteins in FOXJ1 regulatory network.

IIP-effector Category	Gene Name	^1^IIP category	^2^GO association (Ciliary)	^3^Comment
IIP directly regulated by FOXJ1	ATXN1	HUB and BP and CP		
	EEF1A1	HUB and BP and CP		Associated with BBSome proteins (Figure 4), could be involved in cargo trafficking to cilia
	FKBP5	HUB & BP	Protein folding (GO) [GO:0006457∼protein folding]	
	PIAS4	HUB and CP		PIAS4 regulates SUMOylation of Glis2/NPHP7, which is a transcriptional regulator mutated in type 7 nephronophthisis (Ramachandran et al., 2015)
	PLSCR1	HUB and BP		Cilia phenotype defects occur upon knockdown (Choksi et al., 2014a)
	SOCS3	HUB and CP		
	SSX2IP	HUB and CP	Cilium morphogenesis (GO) [GO:0042384∼cilium assembly, GO:0060271∼ cilium morphogenesis, GO:0035735∼intraciliary transport involved in cilium morphogenesis	SSX2IP targets Cep290 to the ciliary transition zone. Cep290 takes a central role in gating proteins to the ciliary compartment (Klinger et al., 2014)
	STOM	HUB and BP and CP		
	SYNCRIP	HUB and BP		
	MEOX2	HUB and BP and CP		
	NLGN3	HUB and BP		
	FAM19A3	HUB and BP		
IIP indirectly regulated by FOXJ1	APOE	HUB and CP	Cytoskeleton organization (GO)[GO:0007010∼ cytoskeleton organization]	
	DLG4	HUB and BP	Establishment or maintenance of epithelial cell apical/basal polarity (GO) [GO:0045197]	
FOXJ1 directly regulated IIP showing differential expression (mRNA) in PCD	ACTN2	CP and GNPP	Actin filament organization (GO)[GO:0051695∼actin filament uncapping, GO:0005884∼actin filament], Cytoskeleton organization (GO) [GO:0005856∼cytoskeleton]	RhoA dependent actin remodeling for establishment of an apical web-like structure of actin for basal body docking and axoneme growth (Kohli et al., 2017)
	CASP8	HUB and BP and CP		
IIP showing differential expression (mRNA) in PCD	BTRC	HUB and BP and CP	Protein ubiquitination (GO)[GO:0000209∼protein polyubiquitination, GO:0006511∼ubiquitin-dependent protein catabolic process, GO:0043161∼proteasome-mediated ubiquitin-dependent protein catabolic process], Cell cycle (GO)[GO:0051726∼regulation of cell cycle]	
	HSP90AA1	HUB and BP and CP	Protein folding (GO)[GO:0006457∼protein folding]	Participates in cilium assembly according to Reactome database (Croft et al., 2014; Fabregat et al., 2018)
	TERF1	HUB and CP		
	CDC42	HUB & CP	Establishment or maintenance of cell polarity (GO)[GO:0007163∼establishment or maintenance of cell polarity], Small GTPase mediated signal transduction (GO) [GO:0007264∼small GTPase mediated signal transduction], Cytoskeleton organization (GO)[GO:0030036∼actin cytoskeleton organization], Actin filament organization (GO) [GO:0051017∼actin filament bundle assembly]	Cdc42 docks vesicles carrying ciliary proteins and localizes the exocyst to primary cilia. CDC42 deficiency results in deranged ciliogenesis and polycystic kidney disease (Choi et al., 2013).

*The identified IIP-effector proteins that figure at the interface of the FOXJ1 regulatory network and the associated PPIN are listed here. The possible roles of some of these IIP-effectors and their primary interactors in cilia are also outlined. ^1^IIP category: Different graph theory metric categories (e.g., hubs: HUB, central proteins: CP, bottleneck proteins: BP and global network perturbing proteins: GNPP) that have identified the protein as topologically important. ^2^GO association (Ciliary): Predicted GO association of IIP-effector. ^3^Comment: Reports whether this protein has been previously associated with cilia assembly or function.*

**FIGURE 4:**
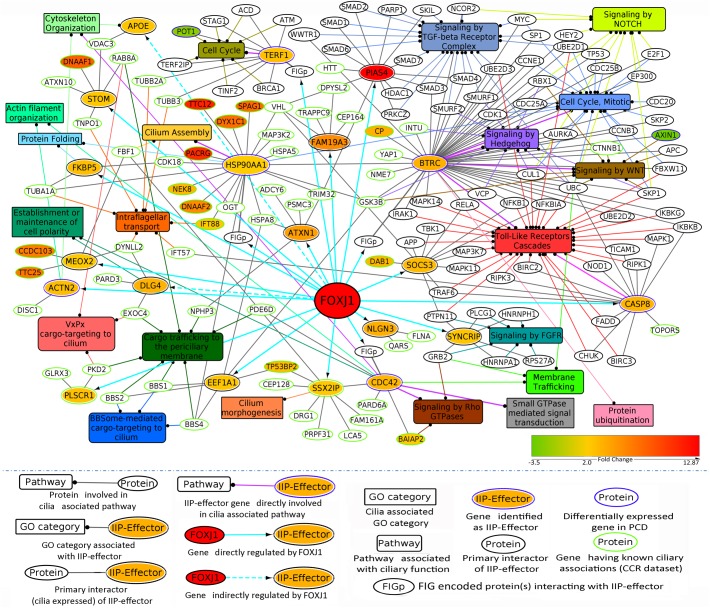
IIP-effectors in FOXJ1 regulatory network and their probable ciliary associations. Probable cilia associated pathways or processes that the IIP-effectors and their network interactors may participate in were determined with the help of pathway enrichment and GO analysis and such possible ciliary role(s) of each IIP-effector is depicted. The edge color denotes the ciliary process(es) or pathway(s) the gene/protein is/are associated with. Fold changes in genes differentially expressed in Choksi et al. expression study (Choksi et al., 2014a) or PCD case study (Geremek et al., 2014) are mapped onto the protein nodes.

Previously, we have determined that the regulatory network proteins forming a part of the inter-connected module interact primarily with signaling proteins in motile ciliated cells (Figure 3B). Therefore, in order to determine which cellular pathways/processes these IIP-effectors might be participating in within the ciliary interactome, we have again utilized the concept of ‘guilt by association.’ GO mapping suggested the involvement of DLG4 and CDC42 in maintenance of cell polarity (establishment or maintenance of epithelial cell apical/basal polarity [GO: 0045197], establishment or maintenance of cell polarity [GO: 0007163], respectively) (Table 2 and Figure 4). Moreover, the pathway enrichment analysis revealed the probable existence of a number of ‘cilia associated signaling pathways’ among IIP-effectors and their primary interactors. Further, based on this analysis, we could identify topologically important signaling proteins in the FOXJ1 regulatory network which are essentially IIP-effectors that were found to be related to or had involvement in some ‘cilia associated signaling pathways.’ In this respect, FOXJ1 effector proteins SYNCRIP and BTRC participate in pathways that regulate motile cilium development like cell cycle, Fgfr, Wnt and Notch signaling (Supplementary Table 6 and Figure 4). CASP8, SOCS3, BTRC, PIAS4 (IIP-effector) and their primary interactors might participate in pathways implicated in primary cilium development and function like TGF-beta, Hedgehog and Toll-like receptor signaling (Supplementary Table 6 and Figure 4). Thus, it appears that these pathways could also be important in motile cilia development and/or function. Importantly, different FOXJ1 regulatory network genes either code for topologically important signaling proteins (CASP8, SOCS3, SYNCRIP) or form extensive cross-talk with topologically important signaling proteins (BTRC, HSP90AA1, CDC42). Further, these ‘protein-pathway’ associations have not previously been studied in the context of ciliogenesis which may be analyzed in further studies.

## Discussion

Here, extensive computational analysis of the FOXJ1 regulatory network and the PPIN associated with it were undertaken to identify essential proteins in the motile cilia interactome and key effector proteins in the FOXJ1 regulatory network that possibly mediate the functional role(s) of FOXJ1. With the help of GO and enrichment analysis of FOXJ1 regulatory network genes, we could identify additional transcription factors and other proteins associated with ciliary structure or motility among FIGp. For instance, directly regulated transcription factors MYCBP and HSBP1 have predicted ciliary associations based on GO analysis, and additional literature studies also indicated that it is likely that MYCBP and HSBP1 may also play a role in ciliogenesis. This is because MYCBP is known to regulate Hedgehog signaling (Lin et al., 2014) and also interacts with A-kinase anchoring proteins (Rao et al., 2016) that are involved in regulating dynein-driven motility, and HSBP1 may be involved in Wnt signaling pathway (Eroglu et al., 2014). Similarly, indirectly regulated genes, ATF5 and CHD4 that participate in maintaining centrosome integrity (Sillibourne et al., 2007; Madarampalli et al., 2015), could also have additional ciliary roles as transcription factors in cilia [defects in centrosome structure or function may lead to ciliopathies; Bettencourt-Dias et al., 2011)]. We observed that the annotated transcriptionally regulated proteins (∼24% of FIGp) mainly comprised of ciliary structural component proteins, mutations in which may be associated with ciliary ultrastructure defects occurring in PCD. However, the other FIGp could also be involved in regulating the assembly or function of cilia in association with other proteins expressed in ciliating cells. In order to identify possible essential proteins for motile cilia development or function, we have studied the PPIN associated with FOXJ1 regulatory network proteins with the assumption that it represents the motile cilia interactome. A number of IIP (121) were identified with the help of an *in silico* node deletion analysis and standard graph theory measures computed based on degree, shortest path and centrality. Furthermore, 33 IIP had previously reported ciliary roles, and it is likely that such topologically important proteins participating in multiple signaling pathways, cell cycle, generic transcription, developmental biology etc. may act as essential proteins in cilia development or function.

Interestingly, 120 IIP along with 246 FIGp form an inter-connected module in the ciliary interactome. Moreover, genes may be associated with a condition based on their differential expression under a diseased state. Thus, differentially expressed genes occurring in PCD patients may be considered as genes associated with motile cilia biogenesis or function. Similarly, genes that are differentially expressed in *in vitro* model systems wherein motile ciliogenesis is perturbed could also be associated with motile cilia biogenesis or function. We have mapped such associations based on expression analysis (Edgar et al., 2002; Choksi et al., 2014a; Geremek et al., 2014) onto our predicted motile cilia interactome. These associated genes (proteins) figure at the interface of the FOXJ1 regulatory network and the associated protein interaction network, and we have classified such IIP as important effector proteins in the FOXJ1 regulatory network. 16 FOXJ1 regulated IIP-effectors share extensive connections with the FOXJ1 regulatory network proteins and some cilia specific PPIN proteins. Subsequently, we have tried to establish the most likely roles of these IIP-effectors in ciliary biology based on the assumption that interacting proteins may share similar cellular functions (‘guilt by association’). Pathway enrichment analysis elucidated that some of these IIP-effectors act as signaling proteins. The IIP-effectors and its interacting partners in the interaction module are particularly involved in Wnt, Notch, Fgfr, Hedgehog, Tgf-beta and Toll-like receptor signaling pathways downstream of FOXJ1 activation. This is in accordance with previous reports wherein Notch, Wnt and Fgf signaling pathways have been shown to regulate processes like left-right patterning, cilia length or number in motile cilia bearing cells (Neugebauer et al., 2009; Lopes et al., 2010; Caron et al., 2012). It is likely that the ‘topologically important signaling proteins’ form a crucial link between the FOXJ1 regulatory and cilia biogenesis associated signaling components in the motile cilium. In particular, BTRC and CASP8 (PCD associated IIP-effector), along with their primary interactors in the ciliary interactome, are possibly involved in mediating Toll-like receptor signaling. Moreover, PCD patients generally are susceptible to recurrent respiratory infections (Alanin et al., 2015) which have been attributed to impaired mucociliary clearance due to cilia motility defects. However, these patients may additionally have impaired TLR signaling that mediate innate immune responses (Kawasaki and Kawai, 2014) or innate immune response pathways due to defects in PCD associated IIP-effectors or their interactome. Other IIP and FIGp also have possible involvement in innate immune responses and Toll-like receptor signaling cascades. Further, IIP-effectors like EEF1A1 and DLG4 were found to be related to well-known processes involved in ciliary biology like maintenance of cell polarity and intra-flagellar transport. EEF1A1 (directly regulated IIP-effector), a small GTPase protein, is likely to be involved in intra-flagellar transport because of its interactions with multiple BBSome component proteins and it has also been reported as an intra-flagellar transport cargo protein (Engel et al., 2012). Likewise, the indirectly regulated IIP-effector DLG4 may be involved in establishing or maintaining apico-basal polarity of cells during ciliogenesis (as predicted by our GO mapping). Therefore, based on cilia associated expression analysis, literature studies, GO and pathway analysis we could rationalize the involvement of the identified topologically important effector proteins in cilia biogenesis or function. Additional experimental studies will help establish their causal link to PCD.

## Conclusion

In conclusion, by analyzing the FOXJ1 associated motile cilia interactome comprised of predicted PPIN of the FOXJ1 regulatory network proteins, we have identified topologically important effector proteins in the motile cilia interactome and FOXJ1 regulatory network. Moreover, we have rationalized their possible roles in ciliary biology with the help of GO and enrichment analysis. We propose that defects in the function(s) of such essential genes may be associated with impaired ciliary development or function, and this list of genes will be useful for screening and diagnosis of novel PCD associated mutations in the future.

## Data Availability

Expression analysis datasets are available in a publicly accessible repository. Choksi et al. (2014a) dataset utilized in this study can be found at Array Express (https://www.ebi.ac.uk/arrayexpress/) under accession number E-MTAB-2815 and the PCD case study dataset can be accessed at GEO (https://www.ncbi.nlm.nih.gov/geo/) under accession number GSE25186. The raw data [collated ciliary resource (prepared from ciliary reference databases and other literature studies)] supporting the conclusions of this manuscript will be made available by the authors, without undue reservation, to any qualified researcher. All other relevant data generated/analyzed for this study are included in the manuscript and the Supplementary Files.

## Author Contributions

SC, SR, and IM formulated the study design. IM performed the experiments. SC, IM, and SR analyzed and interpreted the results. IM, SR, and SC prepared the manuscript. All authors read and approved the final version of the manuscript.

## Conflict of Interest Statement

The authors declare that the research was conducted in the absence of any commercial or financial relationships that could be construed as a potential conflict of interest.
