# Supplementary material for: Identification of Important Effector Proteins in the FOXJ1 Transcriptional Network Associated With Ciliogenesis and Ciliary Function
Source: Front Genet. 2019 Mar 1;10:23. doi: 10.3389/fgene.2019.00023 (PMC6405523; doi:10.3389/fgene.2019.00023)
Supplement: Supplementary file 1 [file Data_Sheet_1.zip › Supplementary Material-Feb20.docx]

Supplementary Material

**Identification of important effector proteins in the FOXJ1 transcriptional network associated with ciliogenesis and ciliary function**

**Ishita Mukherjee^1^, Sudipto Roy^2,3,4*^, and Saikat Chakrabarti^1*^**

*** Correspondence:** Saikat Chakrabarti: [saikat@iicb.res.in](mailto:saikat@iicb.res.in), Sudipto Roy: sudipto@imcb.a-star.edu.sg

# Supplementary Figures and Tables

## Supplementary Figures

**
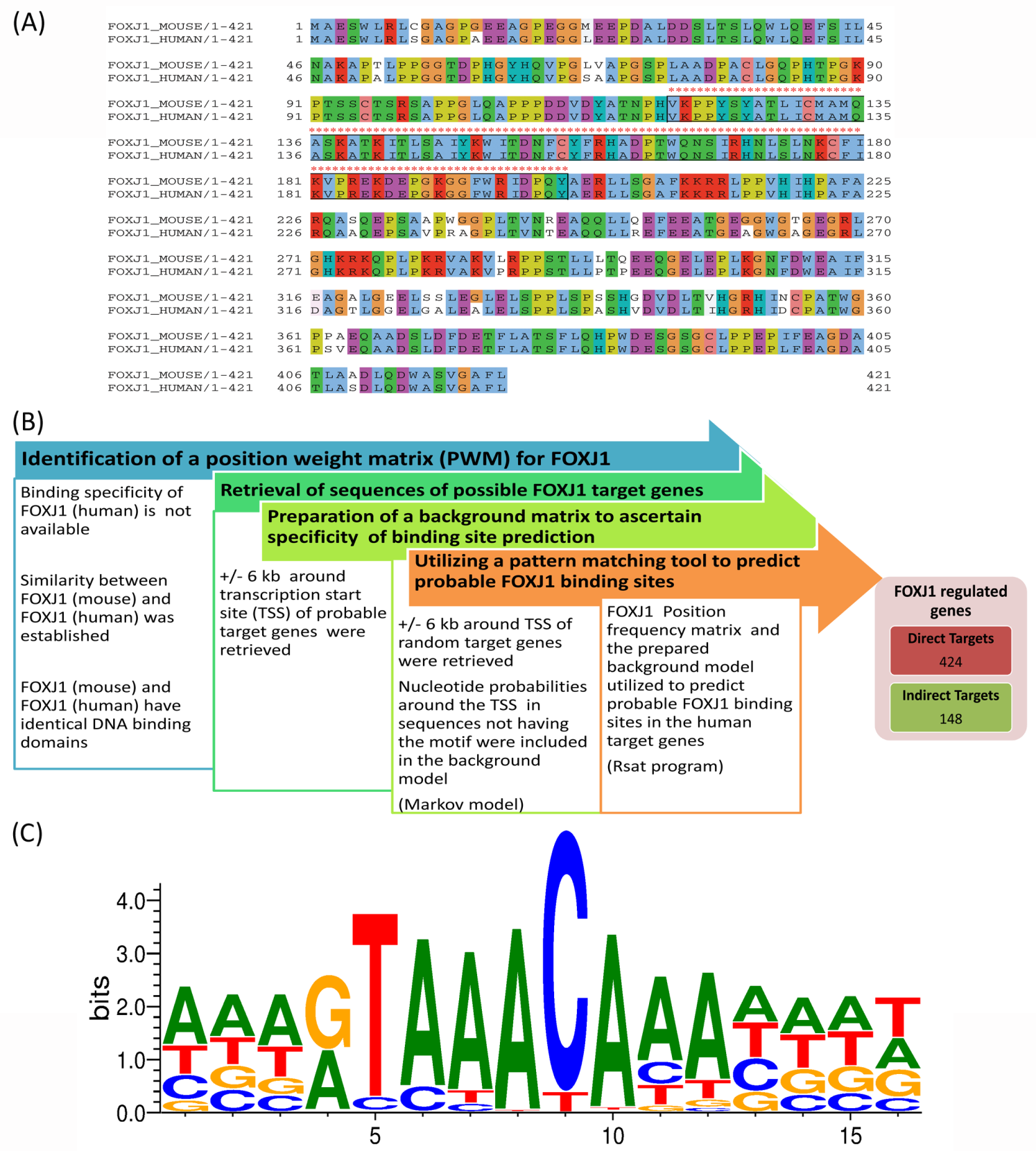
Supplementary Figure 1: Prediction of FOXJ1 regulatory network.** (A) Sequence alignment depicting the evolutionary conservation between the DNA binding domain of FOXJ1 from *Homo sapiens* and *Mus musculus* (denoted by *). (B) Outline of the methodology utilized to identify directly regulated genes by FOXJ1. (C) Logo plot of predicted human FOXJ1 binding sites among FOXJ1 regulated genes.


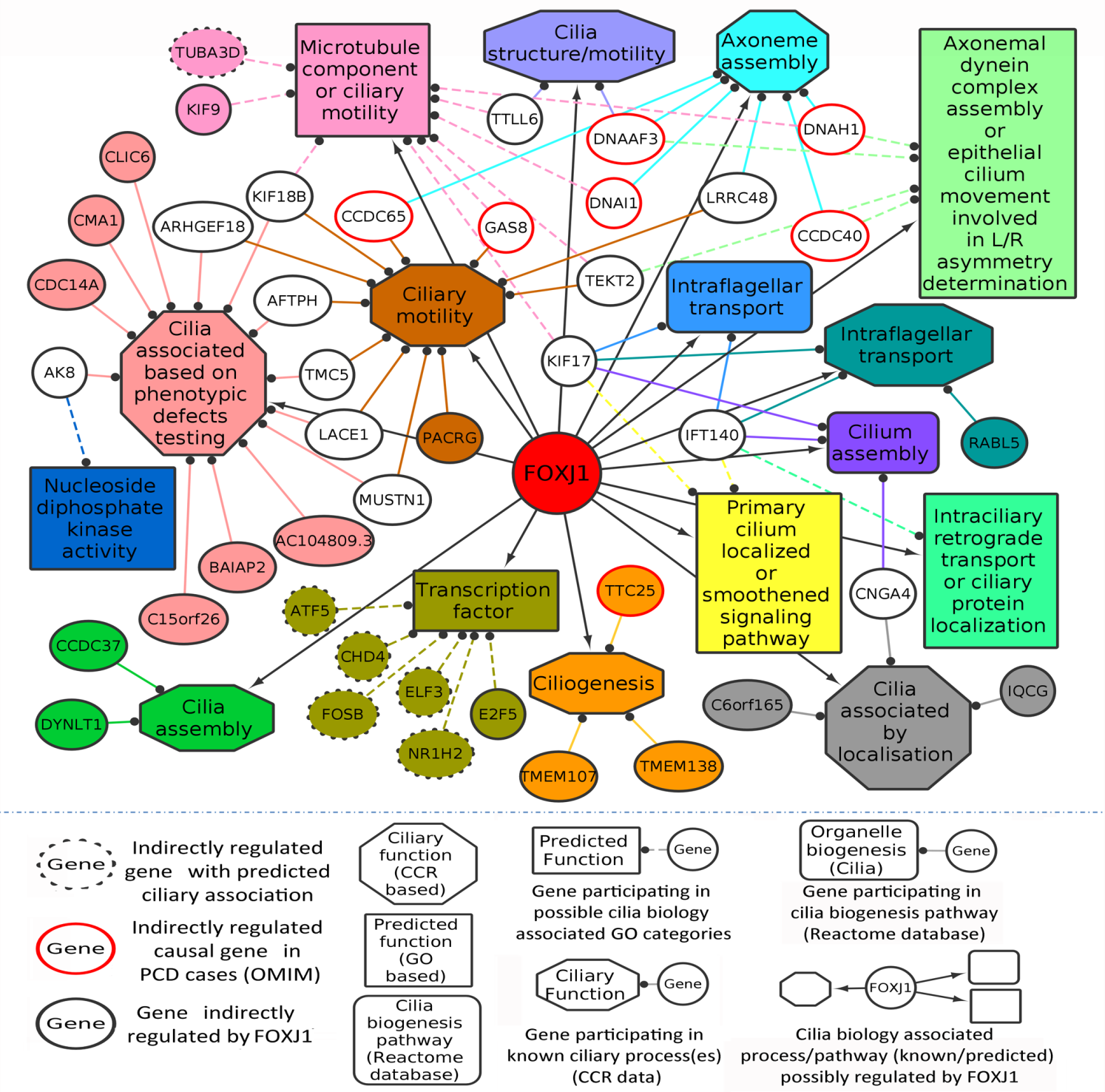


**Supplementary Figure 2: Indirectly regulated genes in predicted FOXJ1 gene regulatory network and possible ciliary processes mediated by them.** FOXJ1 also regulates ciliary assembly or motility via some indirectly regulated genes. Such indirectly regulated genes having ciliary roles, either based on the collated ciliary resource dataset or predictions from gene ontology analysis, are depicted here. The genes and edges are color coded to denote the processes they regulate or are involved in (genes associated with more than one process are not colored).

**
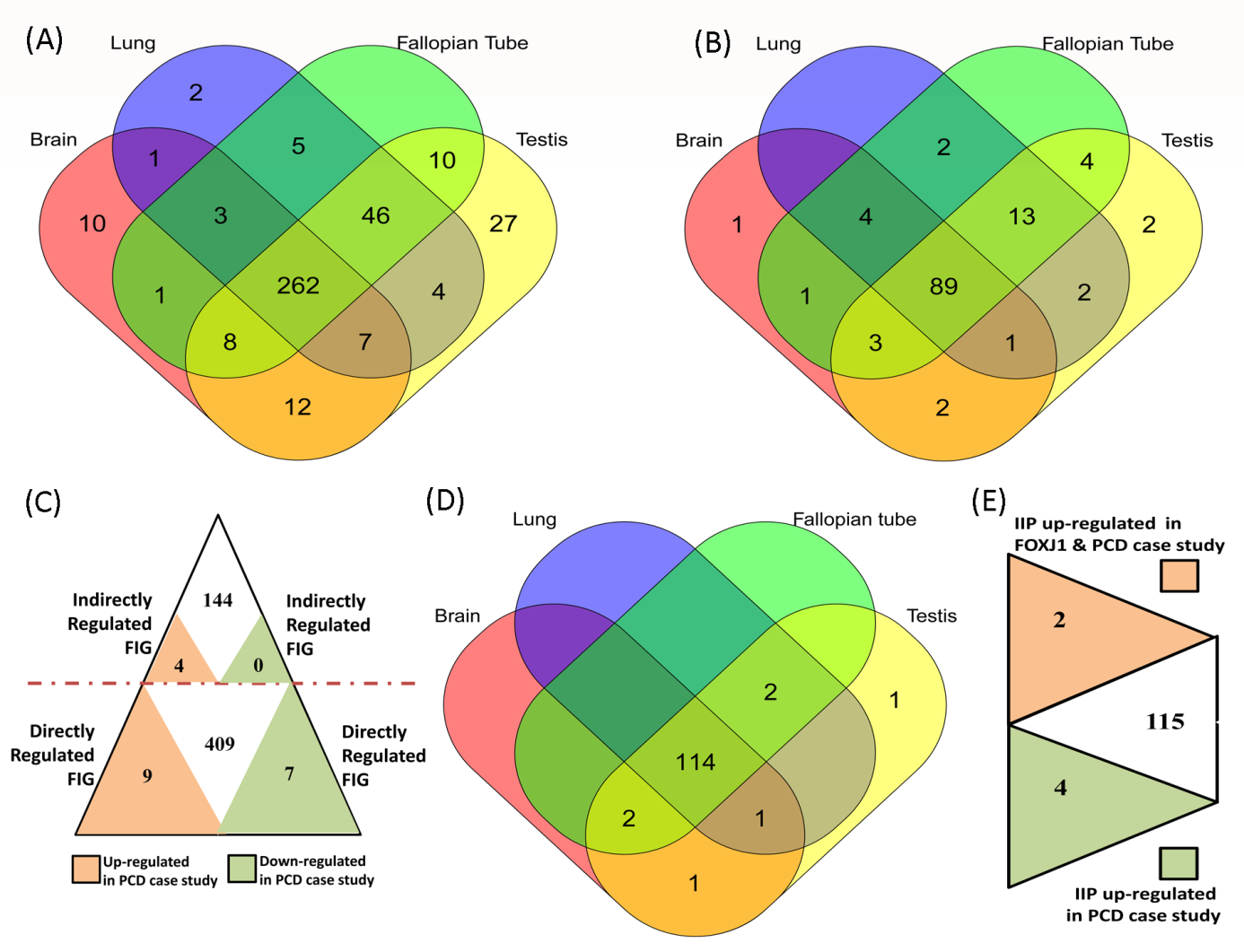
**

**Supplementary Figure 3: Cilia associated expression analysis for FOXJ1 regulatory network genes and IIP.** (A) The Venn diagram depicts the expression pattern of FOXJ1 directly regulated genes among multiple motile ciliated tissues based on expression data collected from the Human Protein Atlas (Uhlén et al., 2015; Thul et al., 2017; Uhlen et al., 2010). (B) The Venn diagram depicts the prevalence of FOXJ1 indirectly regulated genes in different tissues based on expression data collected from the Human Protein Atlas (Uhlén et al., 2015; Thul et al., 2017; Uhlen et al., 2010). (C) The number of FOXJ1 regulatory network genes that could be associated with PCD based on differential expression analysis of bronchial biopsies from PCD patients is outlined. (D) The Venn diagram depicts the expression pattern of IIP among multiple motile ciliated tissues based on expression data in the Human Protein Atlas (Uhlén et al., 2015; Thul et al., 2017; Uhlen et al., 2010). (E) The number of IIP that may be associated with PCD based on differential expression analysis of PCD tissue samples. Abbreviations: PCD: primary ciliary dyskinesia, IIP: important interacting proteins.

## Supplementary Tables

**Supplementary Table 1:** **Genes directly regulated by FOXJ1**. Genes directly regulated by FOXJ1 having possible FOXJ1 binding motif(s) in +/- 6kb of their transcription start sites are listed here. (Attached .xlsx file, note that table footnotes are included in the excel file itself)

**Supplementary Table 2:** **Predicted regulatory network of FOXJ1 regulated genes.** Genes found to be directly or indirectly regulated by FOXJ1 and their assigned ciliary roles based on collated ciliary resource (CCR) are tabulated here. Additional details regarding the regulatory network genes based on GO analysis, disease association, conservation analysis and cilia associated expression analysis have also been included. (Attached .xlsx file, note that table footnotes are included in the excel file itself)

**Supplementary Table 3:** **Predicted ciliary associations for FOXJ1 regulatory network genes based on Gene Ontology (GO) analysis.** Statistics for predicted ciliary functions of FOXJ1 regulatory network genes based on FGNet (Aibar et al., 2015) functional annotation clustering of FIG mapped to different GO categories are shown here. (Attached .xlsx file, note that table footnotes are included in the excel file itself)

**Supplementary Table 4:** **Important interacting proteins (IIP) in the FIG-sub-network identified based on IIP analysis.** Proteins identified as hub, bottleneck, central, local network perturbing or global network perturbing and in turn as IIP with the help of multiple graph theory measures formulated on degree, shortest path and centrality are listed here.

Abbreviations: BP - Bottleneck protein, CP - Central protein, LNPP - Local network perturbing protein, GNPP- Global network perturbing protein (Attached .xlsx file, note that table footnotes are included in the excel file itself)

**Supplementary Table 5: Ciliary roles that FOXJ1 regulatory network proteins may participate in along with important interacting proteins (IIP).** Significantly enriched pathways, considering FOXJ1 regulatory network proteins and IIP as participating clusters of proteins in each pathway, have been elucidated here. Note: All enriched pathways (child processes) were found to participate in enriched parent pathways. Thus only parent pathways have been included here. (Attached .xlsx file, note that table footnotes are included in the excel file itself)

**Supplementary Table 6: Cilia-associated pathways possibly mediated by IIP-effectors and their interacting network proteins.** Enriched cilia associated signaling pathways and their parent Reactome pathways (Fabregat et al., 2018; Croft et al., 2014) in which IIP-effectors and their interacting proteins are likely to be involved in have been exemplified here. (Attached .xlsx file, note that table footnotes are included in the excel file itself)

**References:**

Absalon, S., Blisnick, T., Kohl, L., Toutirais, G., Doré, G., Julkowska, D., et al. (2008). Intraflagellar transport and functional analysis of genes required for flagellum formation in Trypanosomes. Mol Biol Cell.;19(3), 929–944. doi: 10.1091/mbc.E07-08-0749

Adhiambo, C., Blisnick, T., Toutirais, G., Delannoy, E., Bastin, P. (2009) A novel function for the atypical small G protein Rab-like 5 in the assembly of the trypanosome flagellum. J Cell Sci.;122(Pt 6):834-41. doi: 10.1242/jcs.040444.

Aibar, S., Fontanillo, C., Droste, C. and De Las Rivas, J. (2015) Functional Gene Networks: R/Bioc package to generate and analyze gene networks derived from functional enrichment and clustering. Bioinformatics;31(10), 1686-1688.  doi: 10.1093/bioinformatics/btu864

Amberger, J.S., Bocchini, C.A., Schiettecatte, F., Scott, A.F., Hamosh, A. (2015) OMIM.org: Online Mendelian Inheritance in Man (OMIM®), an online catalog of human genes and genetic disorders. Nucleic Acids Res.; 43:D789-798. doi: 10.1093/nar/gku1205

Arnaiz, O., Cohen, J., Tassin, A.M., Koll, F. (2014) Remodeling Cildb, a popular database for cilia and links for ciliopathies. Cilia; 3:9. doi:10.1186/2046-2530-3-9

Arnaiz, O., Malinowska, A., Klotz, C., Sperling, L, Dadlez, M., Koll, F., et al. (2009) Cildb: a knowledgebase for centrosomes and cilia. Database (Oxford),bap022. doi:10.1093/database/bap022

Austin-Tse, C., Halbritter, J., Zariwala, M. A., Gilberti, R. M., Gee, H. Y., Hellman, N., et al. (2013). Zebrafish ciliopathy screen plus human mutational analysis identifies C21orf59 and CCDC65 defects as causing primary ciliary dyskinesia. Am J Hum Genet.; 93(4), 672–686. doi: 10.1016/j.ajhg.2013.08.015

Beales, P.L., Bland, E., Tobin, J.L., Bacchelli, C., Tuysuz, B., Hill, J. et al. (2007) IFT80, which encodes a conserved intraflagellar transport protein, is mutated in Jeune asphyxiating thoracic dystrophy. Nat Genet.;39(6):727-9. doi: 10.1038/ng2038

Becker-Heck, A., Zohn, I., Okabe, N., Pollock, A., Lenhart, K. B., Sullivan-Brown, J., et al. (2011). The coiled-coil domain containing protein CCDC40 is essential for motile cilia function and left-right axis formation. Nat Genet.;43(1), 79–84. doi: 10.1038/ng.727

Bershteyn, M., Atwood, S. X., Woo, W. M., Li, M., & Oro, A. E. (2010). MIM and Cortactin antagonism regulates ciliogenesis and Hedgehog signaling. Dev Cell. 19(2), 270–283. doi: 10.1016/j.devcel.2010.07.009

Bhowmick, R., Li, M., Sun, J., Baker, S. A., Insinna, C., & Besharse, J. C. (2009). Photoreceptor IFT omplexes ontaining Chaperones, Guanylyl Cyclase 1, and Rhodopsin. Traffic (Copenhagen, Denmark), 10(6), 648–663. doi: 10.1111/j.1600-0854.2009.00896.x

Boldt, K., van Reeuwijk, J., Lu, Q., Koutroumpas, K., Nguyen, T.M., Texier, Y., et al. (2016) An organelle-specific protein landscape identifies novel diseases and molecular mechanisms. Nat Commun.;7:11491.doi:10.1038/ncomms11491

Bontems, F., Fish, R. J., Borlat, I., Lembo, F., Chocu, S., Chalmel, F., Lane, L. (2014). C2orf62 and TTC17 are involved in actin organization and ciliogenesis in zebrafish and human. PLoS ONE; 9(1), e86476. doi : 10.1371/journal.pone.0086476

Cao, Y., Semanchik, N., Lee, S. H., Somlo, S., Barbano, P. E., Coifman, R., & Sun, Z. (2009). Chemical modifier screen identifies HDAC inhibitors as suppressors of PKD models. Proc Natl Acad Sci U S A.; 106(51), 21819–21824. doi: 10.1073/pnas.0911987106

Caron, A., Xu, X., Lin, X. (2012) Wnt/beta-catenin signaling directly regulates Foxj1 expression and ciliogenesis in zebrafish Kupffer's vesicle. Development.; 139:514-524. doi:10.1242/dev.071746

Castleman, V. H., Romio, L., Chodhari, R., Hirst, R. A., de Castro, S. C. P., Parker, K. A., et al. (2009). Mutations in radial spoke head protein genes RSPH9 and RSPH4A cause primary ciliary dyskinesia with central-microtubular-pair abnormalities. Am J Hum Genet.; 84(2), 197–209. doi: 10.1016/j.ajhg.2009.01.011

Chandrasekar, G., Vesterlund, L., Hultenby, K., Tapia-Páez, I., & Kere, J. (2013). The zebrafish orthologue of the dyslexia candidate gene DYX1C1 is essential for cilia growth and function. PLoS ONE;8(5), e63123. doi: 10.1371/journal.pone.0063123

Chang, J., Baloh, R. H., & Milbrandt, J. (2009). The NIMA-family kinase Nek3 regulates microtubule acetylation in neurons. J Cell Sci.;122(13), 2274–2282. doi: 10.1242/jcs.048975

Choi, S. Y., Chacon-Heszele, M. F., Huang, L., McKenna, S., Wilson, F. P., Zuo, X., & Lipschutz, J. H. (2013). Cdc42 deficiency causes ciliary abnormalities and cystic kidneys.  J Am Soc Nephrol.; 24(9), 1435–1450. doi: 10.1681/ASN.2012121236

Choksi, S.P., Babu D., Lau D., Yu X., and Roy S. (2014). Systematic discovery of novel ciliary genes through functional genomics in the zebrafish. Development; 141:3410-19. doi:10.1242/dev.108209

Chung, M.-I., Kwon, T., Tu, F., Brooks, E. R., Gupta, R., Meyer, M., et al. (2014). Coordinated genomic control of ciliogenesis and cell movement by RFX2. eLife; 3, e01439. doi: 10.7554/eLife.01439

Clement, C.A., Ajbro, K.D., Koefoed, K., Vestergaard, M.L., Veland, I.R., Henriques de Jesus M.P. et al. (2013) TGF-beta signaling is associated with endocytosis at the pocket region of the primary cilium. Cell Rep.; 3:1806-1814.doi:10.1016/j.celrep.2013.05.020

Colantonio, J. R., Vermot, J., Wu, D., Langenbacher, A. D., Fraser, S., Chen, J.-N., & Hill, K. L. (2009). The dynein regulatory complex is required for ciliary motility and otolith biogenesis in the inner ear. Nature; 457(7226), 10.1038/nature07520. doi: 10.1038/nature07520

Conductier, G., Martin, A. O., Risold, P.-Y., Jego, S., Lavoie, R., Lafont, C., et al. (2013). Control of ventricular ciliary beating by the melanin concentrating hormone-expressing neurons of the lateral hypothalamus: a functional imaging survey. Front Endocrinol (Lausanne).; 4, 182. doi: 10.3389/fendo.2013.00182

Conroy, P. C., Saladino, C., Dantas, T. J., Lalor, P., Dockery, P., & Morrison, C. G. (2012). C-NAP1 and rootletin restrain DNA damage-induced centriole splitting and facilitate ciliogenesis. Cell Cycle;11(20), 3769–3778. doi: 10.4161/cc.21986

Croft, D., Mundo, A. F., Haw, R., Milacic, M., Weiser, J., Wu, G., et al. (2014). The Reactome pathway knowledgebase. Nucleic Acids Res.; 42(Database issue), D472–D477. doi: 10.1093/nar/gkt1102

Danescu, S., Has, C., Baican, C. , Müller, T. and Baican, A. (2018) A novel IKBKG mutation in a patient with incontinentia pigmenti and features of hepatic ciliopathy. Australas J Dermatol.; ;59(4):e262-e265.doi:10.1111/ajd.12805

Delaval, B., Covassin, L., Lawson, N. D., & Doxsey, S. (2011). Centrin depletion causes cyst formation and other ciliopathy-related phenotypes in zebrafish. Cell Cycle;10(22), 3964–3972. doi: 10.4161/cc.10.22.18150

Dong, F., Shinohara, K., Botilde, Y., Nabeshima, R., Asai, Y., Fukumoto, A., et al. (2014). Pih1d3 is required for cytoplasmic preassembly of axonemal dynein in mouse sperm. J Cell Biol.;204(2), 203–213. doi: 10.1083/jcb.201304076

Epting, D., Slanchev, K., Boehlke, C., Hoff, S., Loges, N.T., Yasunaga, T. et al. (2015) The Rac1 regulator ELMO controls basal body migration and docking in multiciliated cells through interaction with Ezrin. Development.;142:174-184. doi: 10.1242/dev.112250

Fabregat, A., Jupe, S., Matthews, L., Sidiropoulos, K., Gillespie, M., Garapati, P., et al. (2018). The Reactome Pathway Knowledgebase. Nucleic Acids Res.; 46 (Database issue), D649–D655. doi: 10.1093/nar/gkx1132

Fernandez-Gonzalez, A., Kourembanas, S., Wyatt, T. A., & Mitsialis, S. A. (2009). Mutation of murine adenylate kinase 7 underlies a primary ciliary dyskinesia phenotype. Am J Respir Cell Mol Biol.; 40(3), 305–313. doi: 10.1165/rcmb.2008-0102OC

Gerdes, J.M., Liu, Y., Zaghloul, N.A., Leitch, C.C., Lawson, S.S., Kato, M. et al. (2007) Disruption of the basal body compromises proteasomal function and perturbs intracellular Wnt response. Nat Genet.; 39:1350-1360. doi: 10.1038/ng.2007.12

Geremek, M., Ziętkiewicz, E., Bruinenberg, M., Franke, L., Pogorzelski, A., Wijmenga, C., et al (2014). Ciliary genes are down-regulated in bronchial tissue of primary ciliary dyskinesia patients. PLoS ONE.; 9(2), e88216. doi: 10.1371/journal.pone.0088216

Glazer, A., Wilkinson, A., Backer, C. B., Lapan, S., Gutzman, J. H., Cheeseman, I. M., & Reddien, P. W. (2010). The Zn Finger protein Iguana impacts Hedgehog signaling by promoting ciliogenesis. Dev Biol.;337(1), 148–156. doi: 10.1016/j.ydbio.2009.10.025

Gorivodsky, M., Mukhopadhyay, M., Wilsch-Braeuninger, M., Phillips, M., Teufel, A., Kim, C., et al. (2009). Intraflagellar Transport Protein 172 is essential for primary cilia formation and plays a vital role in patterning the mammalian brain. Dev Biol.;325(1), 24–32. doi: 10.1016/j.ydbio.2008.09.019

Gupta, G.D., Coyaud, E., Goncalves, J., Mojarad, B.A., Liu, Y., Wu, Q., et al. (2015) A Dynamic Protein Interaction Landscape of the Human Centrosome-Cilium Interface. Cell; 163:1484-1499. doi:10.1016/j.cell.2015.10.065

Han, Y. M., Kang, G. M., Byun, K., Ko, H. W., Kim, J., Shin, M.-S. et al. (2014). Leptin-promoted cilia assembly is critical for normal energy balance. J Clin Invest.;124(5), 2193–2197. doi:.10.1172/JCI69395

Hashimoto, H., Miyamoto, R., Watanabe, N., Shiba, D., Ozato, K., Inoue, C., et al. (2009). Polycystic kidney disease in the Medaka (*Oryzias latipes*) pc mutant caused by a mutation in the Gli-Similar3 (glis3) gene. PLoS ONE; 4(7), e6299. doi: 10.1371/journal.pone.0006299

Hayes, J. M., Kim, S. K., Abitua, P. B., Park, T. J., Herrington, E. R., Kitayama, A., et al. (2007). Identification of novel ciliogenesis factors using a new in vivo model for mucociliary epithelial development. Dev Biol.;312(1), 115–130. doi: 10.1016/j.ydbio.2007.09.01

Herrero, J., Muffato, M., Beal, K., Fitzgerald, S., Gordon, L., Pignatelli, M., et al. (2016). Ensembl comparative genomics resources. Database (Oxford)*;*bav096. doi:10.1093/database/bav096

Hjeij, R., Lindstrand, A., Francis, R., Zariwala, M. A., Liu, X., Li, Y.et al. (2013). ARMC4 mutations cause primary ciliary dyskinesia with randomization of left/right body asymmetry. Am J Hum Genet.;93(2), 357–367. doi: 10.1016/j.ajhg.2013.06.009

Hori, A., Ikebe, C., Tada, M., & Toda, T. (2014). Msd1/SSX2IP-dependent microtubule anchorage ensures spindle orientation and primary cilia formation. EMBO Rep.; 15(2), 175–184. doi: 10.1002/embr.201337929

Hurtado, L., Caballero, C., Gavilan, M. P., Cardenas, J., Bornens, M., & Rios, R. M. (2011). Disconnecting the Golgi ribbon from the centrosome prevents directional cell migration and ciliogenesis. J Cell Biol.; 193(5), 917–933. doi: 10.1083/jcb.201011014

Insinna, C., Pathak, N., Perkins, B., Drummond, I., & Besharse, J. C. (2008). The homodimeric kinesin, Kif17, is essential for vertebrate photoreceptor sensory outer segment development. Dev Biol.;316(1), 160–170. doi:.10.1016/j.ydbio.2008.01.025

Jauregui, A. R., Nguyen, K. C. Q., Hall, D. H., & Barr, M. M. (2008). The *Caenorhabditis elegans* nephrocystins act as global modifiers of cilium structure. J Cell Biol.; 180(5), 973–988. doi: 10.1083/jcb.200707090

Jerber, J., Baas, D., Soulavie, F., Chhin, B., Cortier, E., Vesque, C., et al. (2014) The coiled-coil domain containing protein CCDC151 is required for the function of IFT-dependent motile cilia in animals. Hum Mol Genet.; 23(3):563-77. doi: 10.1093/hmg/ddt445

Kasahara, K., Aoki, H., Kiyono, T., Wang, S., Kagiwada, H., Yuge, M., et al. (2018). EGF receptor kinase suppresses ciliogenesis through activation of USP8 deubiquitinase. Nat Commun.; 9(1):758. doi: 10.1038/s41467-018-03117-y

Kim, J., Lee, J. E., Heynen, S., Suyama, E., Ono, K., Lee, K., et al. (2010). Functional genomic screen for modulators of ciliogenesis and cilium length. Nature.; 464(7291), 1048–1051. doi: 10.1038/nature08895

Klinger, M., Wang, W., Kuhns, S., Bärenz, F., Dräger-Meurer, S., Pereira, G., & Gruss, O. J. (2014). The novel centriolar satellite protein SSX2IP targets Cep290 to the ciliary transition zone. Mol Biol Cell.;25(4), 495–507. doi: 10.1091/mbc.E13-09-0526

Knowles, M. R., Leigh, M. W., Ostrowski, L. E., Huang, L., Carson, J. L., Hazucha, M. J. et al. (2013a) Exome sequencing identifies mutations in CCDC114 as a cause of primary ciliary dyskinesia. Am J Hum Genet.; 92(1), 99–106. doi: 10.1016/j.ajhg.2012.11.003

Knowles, M. R., Ostrowski, L. E., Loges, N. T., Hurd, T., Leigh, M. W., Huang, L., et al. (2013b). Mutations in SPAG1 cause primary ciliary dyskinesia associated with defective outer and inner dynein arms. Am J Hum Genet.; 93(4), 711–720. doi: 10.1016/j.ajhg.2013.07.025

Ko, H. W., Norman, R. X., Tran, J., Fuller, K. P., Fukuda, M., & Eggenschwiler, J. T. (2010). Broad-minded links cell cycle-related kinase to cilia assembly and Hedgehog signal transduction.  Dev Cell.;18(2), 237–247. doi: 10.1016/j.devcel.2009.12.014

Kobayashi, T., Nakazono, K., Tokuda, M., Mashima, Y., Dynlacht, B. D., & Itoh, H. (2017). HDAC2 promotes loss of primary cilia in pancreatic ductal adenocarcinoma. EMBO Rep.; 18(2), 334–343. doi: 10.15252/embr.201541922

Kohli, P., Hohne, M., Jungst, C., Bertsch, S., Ebert, L.K., Schauss, A.C., et al. (2017) The ciliary membrane-associated proteome reveals actin-binding proteins as key components of cilia. EMBO Rep; 18:1521-1535.doi:10.15252/embr.201643846

Kott, E., Duquesnoy, P., Copin, B., Legendre, M., Dastot-Le Moal, F., Montantin, G., et al. (2012). Loss-of-function mutations in LRRC6, a gene essential for proper axonemal assembly of inner and outer dynein arms, cause primary ciliary dyskinesia. Am J Hum Genet.;91(5), 958–964. doi: 10.1016/j.ajhg.2012.10.003

Kott, E., Legendre, M., Copin, B., Papon, J.-F., Dastot-Le Moal, F., Montantin, G., et al. (2013). Loss-of-function mutations in RSPH1 cause primary ciliary dyskinesia with central-complex and radial-spoke defects. Am J Hum Genet.; 93(3), 561–570. doi: 10.1016/j.ajhg.2013.07.013

Kubo, T., Yanagisawa, H.A., Yagi, T., Hirono, M., Kamiya, R. (2010) Tubulin polyglutamylation regulates axonemal motility by modulating activities of inner-arm dyneins. Curr Biol.;20(5):441-5. doi: 10.1016/j.cub.2009.12.058.

Lai, C. K., Gupta, N., Wen, X., Rangell, L., Chih, B., Peterson, A. S., et al. (2011). Functional characterization of putative cilia genes by high-content analysis. Mol Biol Cell.; 22(7), 1104–1119. doi: 10.1091/mbc.E10-07-0596

Lechtreck, K.-F., Delmotte, P., Robinson, M. L., Sanderson, M. J., & Witman, G. B. (2008). Mutations in Hydin impair ciliary motility in mice. J Cell Biol.;180(3), 633–643. doi: 10.1083/jcb.200710162

Lee, J. H., Silhavy, J. L., Lee, J. E., Al-Gazali, L., Thomas, S., Davis, E. E., et al. (2012). Evolutionarily assembled cis-regulatory module at a human ciliopathy locus. Science (New York, N.Y.); 335(6071), 966–969. doi: 10.1126/science.1213506

Lee, L., Campagna, D. R., Pinkus, J. L., Mulhern, H., Wyatt, T. A., Sisson, J. H., et al. (2008). Primary ciliary dyskinesia in mice lacking the novel ciliary protein Pcdp1. Mol Cell Biol.;28(3), 949–957. doi: 10.1128/MCB.00354-07

Liu, X., Bulgakov, O. V., Darrow, K. N., Pawlyk, B., Adamian, M., Liberman, M. C., & Li, T. (2007). Usherin is required for maintenance of retinal photoreceptors and normal development of cochlear hair cells. Proc Natl Acad Sci U S A.;104(11), 4413–4418. doi:10.1073/pnas.0610950104

Loges, N. T., Olbrich, H., Becker-Heck, A., Häffner, K., Heer, A., Reinhard, C., et al. (2009). Deletions and point mutations of LRRC50 cause primary ciliary dyskinesia due to dynein arm defects. Am J Hum Genet.;85(6), 883–889. doi: 10.1016/j.ajhg.2009.10.018

Lutz, M.S., Burk, R.D. (2006) Primary cilium formation requires von hippel-lindau gene function in renal-derived cells. Cancer Res.; 66:6903-6907. doi: 10.1158/0008-5472.CAN-06-0501

Maisonneuve, C., Guilleret, I., Vick, P., Weber, T., Andre, P., Beyer, T. (2009) Bicaudal C, a novel regulator of Dvl signaling abutting RNA-processing bodies, controls cilia orientation and leftward flow. Development;136(17):3019-30. doi: 10.1242/dev.038174.

Malicki, J. J., & Johnson, C. A. (2017). The cilium: cellular antenna and central processing unit. Trends Cell Biol.;27(2), 126–140. doi: 10.1016/j.tcb.2016.08.002

Marley, A., & von Zastrow, M. (2010). DISC1 regulates primary cilia that display specific dopamine receptors. PLoS ONE.;5(5), e10902. doi: 10.1371/journal.pone.0010902

Maskey, D., Marlin, M. C., Kim, S., Kim, S., Ong, E.-C., Li, G., & Tsiokas, L. (2015). Cell cycle-dependent ubiquitylation and destruction of NDE1 by CDK5-FBW7 regulates ciliary length. EMBO J.; 34(19), 2424–2440. doi: 10.15252/embj.201490831

McKusick, V.A. (1998) Mendelian Inheritance in Man. A catalog of human genes and genetic disorders. Baltimore: Johns Hopkins University Press, (12th edition) <https://omim.org/>

Merrill, A. E., Merriman, B., Farrington-Rock, C., Camacho, N., Sebald, E. T., Funari, V. A., et al. (2009). Ciliary abnormalities due to defects in the retrograde transport protein DYNC2H1 in short-rib polydactyly syndrome. Am J Hum Genet.; 84(4), 542–549. doi: 10.1016/j.ajhg.2009.03.015

Merveille, A.-C., Davis, E. E., Becker-Heck, A., Legendre, M., Amirav, I., Bataille, G., et al. (2011). CCDC39 is required for assembly of inner dynein arms and the dynein regulatory complex and for normal ciliary motility in humans and dogs. Nat Genet.;43(1), 72–78. doi:10.1038/ng.726

Mill, P., Lockhart, P. J., Fitzpatrick, E., Mountford, H. S., Hall, E. A., Reijns, M. A. M., et al. (2011). Human and mouse mutations in WDR35 cause short-rib polydactyly syndromes due to abnormal ciliogenesis. Am J Hum Genet.;88(4), 508–515. doi: 10.1016/j.ajhg.2011.03.015

Moore, D. J., Onoufriadis, A., Shoemark, A., Simpson, M. A., zur Lage, P. I., de Castro, S. C., et al. (2013). Mutations in ZMYND10, a gene essential for proper axonemal assembly of inner and outer dynein arms in humans and flies, cause primary ciliary dyskinesia. Am J Hum Genet.;93(2), 346–356. doi: 10.1016/j.ajhg.2013.07.009

Neesen, J., Kirschner, R., Ochs, M., Schmiedl, A., Habermann, B., Mueller, C. et al. (2001) Disruption of an inner arm dynein heavy chain gene results in asthenozoospermia and reduced ciliary beat frequency. Hum Mol Genet.;10(11):1117-28.

Niwa, S., Nakajima, K., Miki, H., Minato, Y., Wang, D., Hirokawa, N. (2012) KIF19A is a microtubule-depolymerizing kinesin for ciliary length control. Dev Cell.;23(6):1167-75 doi: 10.1016/j.devcel.2012.10.016

Olbrich, H., Häffner, K., Kispert, A., Völkel, A., Volz, A., Sasmaz, G. et al. (2002) Mutations in DNAH5 cause primary ciliary dyskinesia and randomization of left-right asymmetry. Nat Genet.;30(2):143-4. doi: 10.1038/ng817

Omran, H., Kobayashi, D., Olbrich, H., Tsukahara, T., Loges, N. T., Hagiwara, H., et al. (2008). Ktu/PF13 is required for cytoplasmic pre-assembly of axonemal dyneins. Nature;456(7222), 611–616. doi: 10.1038/nature07471

Palmer, K. J., MacCarthy-Morrogh, L., Smyllie, N., & Stephens, D. J. (2011). A role for Tctex-1 (DYNLT1) in controlling primary cilium length. Eur J Cell Biol.;90(10), 865–871. doi: 10.1016/j.ejcb.2011.05.003

Pan, J., You, Y., Huang, T., Brody, S.L.(2007) RhoA-mediated apical actin enrichment is required for ciliogenesis and promoted by Foxj1. J Cell Sci;120:1868-1876. doi:10.1242/jcs.005306

Panizzi, J. R., Becker-Heck, A., Castleman, V. H., Al-Mutairi, D., Liu, Y., Loges, N. T., et al (2012). CCDC103 mutations cause primary ciliary dyskinesia by disrupting assembly of ciliary dynein arms. Nat Genet.; 44(6), 714–719. doi: 10.1038/ng.2277

Patzke, S., Redick, S., Warsame, A., Murga-Zamalloa, C. A., Khanna, H., Doxsey, S., & Stokke, T. (2010). CSPP is a ciliary protein interacting with Nephrocystin 8 and required for cilia formation. Mol Biol Cell.; 21(15), 2555–2567. doi: 10.1091/mbc.E09-06-0503

Pazour, G. J., Dickert, B. L., Vucica, Y., Seeley, E. S., Rosenbaum, J. L., Witman, G. B., & Cole, D. G. (2000). Chlamydomonas IFT88 and its mouse homologue, polycystic kidney disease gene Tg737, are required for assembly of cilia and flagella. J Cell Biol.;151(3), 709–718.

Qin, H., Wang, Z., Diener, D., & Rosenbaum, J. (2007). Intraflagellar transport protein 27 is a small G protein involved in cell-cycle control. Curr Biol.;17(3), 193–202. doi: 10.1016/j.cub.2006.12.040

Ramachandran, H., Herfurth, K., Grosschedl, R., Schäfer, T., & Walz, G. (2015). SUMOylation blocks the Ubiquitin-mediated degradation of the nephronophthisis gene product Glis2/NPHP7. PLoS ONE;10(6), e0130275. doi: 10.1371/journal.pone.0130275

Raman, M., Sergeev, M., Garnaas, M., Lydeard, J. R., Huttlin, E. L., Goessling, W. et al. (2015). Systematic VCP-UBXD adaptor network proteomics identifies a role for UBXN10 in regulating ciliogenesis. Nat Cell Biol; 17(10), 1356–1369. doi: 10.1038/ncb3238

Roy, A., Lin, Y.N., Agno, J.E., DeMayo, F.J., Matzuk, M.M. (2007) Absence of tektin 4 causes asthenozoospermia and subfertility in male mice. FASEB J.;21(4):1013-25. doi: 10.1096/fj.06-7035com

Sapiro, R., Kostetskii, I., Olds-Clarke, P., Gerton, G. L., Radice, G. L., & Strauss III, J. F. (2002). Male infertility, impaired sperm motility, and hydrocephalus in mice deficient in sperm-associated antigen 6. Mol Cell Biol.;22(17), 6298–6305. doi: 10.1128/MCB.22.17.6298-6305.2002

Schaub, J. R., & Stearns, T. (2013). The Rilp-like proteins Rilpl1 and Rilpl2 regulate ciliary membrane content. Mol Biol Cell.;24(4), 453–464. doi: 10.1091/mbc.E12-08-0598

Schmid, F.M., Schou, K.B., Vilhelm, M.J., Holm, M.S., Breslin, L., Farinelli, P., et al. (2018) IFT20 modulates ciliary PDGFRα signaling by regulating the stability of Cbl E3 ubiquitin ligases. J Cell Biol.; 217(1):151-161. doi: 10.1083/jcb.201611050.

Schwabe, G.C., Hoffmann, K., Loges, N.T., Birker, D., Rossier, C., de Santi, M.M., Olbrich, H., Fliegauf, M., Failly, M., Liebers, U. et al. (2008) Primary ciliary dyskinesia associated with normal axoneme ultrastructure is caused by DNAH11 mutations. Hum Mutat.;29:289–298 doi: 10.1002/humu.20656

Shnitsar, I., Bashkurov, M., Masson, G. R., Ogunjimi, A. A., Mosessian, S., Cabeza, E. A., et al. (2015). PTEN regulates cilia through Dishevelled. Nat Commun.; 6, 8388. doi: 10.1038/ncomms9388

Sironen, A., Kotaja, N., Mulhern, H., Wyatt, T. A., Sisson, J. H., Pavlik, J. A., et al. (2011). Loss of SPEF2 function in mice results in spermatogenesis defects and primary ciliary dyskinesia. Biol Reprod.;85(4), 690–701. doi: 10.1095/biolreprod.111.091132

Smith, L.A., Bukanov, N.O., Husson, H., Russo, R.J., Barry, T.C., Taylor, A.L. (2006) Development of polycystic kidney disease in juvenile cystic kidney mice: insights into pathogenesis, ciliary abnormalities, and common features with human disease. J Am Soc Nephrol.; 17(10):2821-31. doi: 10.1681/ASN.2006020136

Suizu, F., Hirata, N., Kimura, K., Edamura, T., Tanaka, T., Ishigaki, S. et al (2016). Phosphorylation dependent Akt–Inversin interaction at the basal body of primary cilia. EMBO J.; 35(12), 1346–1363. doi: 10.15252/embj.201593003

Suryavanshi, S., Eddé, B., Fox, L. A., Guerrero, S., Hard, R., Hennessey, T., et al. (2010). Tubulin glutamylation regulates ciliary motility by altering inner dynein arm activity. Curr Biol.; (5), 435–440. doi: 10.1016/j.cub.2009.12.062

Suzuki, T., Miyamoto, H., Nakahari, T., Inoue, I., Suemoto, T., Jiang, B., et al. (2009). Efhc1 deficiency causes spontaneous myoclonus and increased seizure susceptibility. Hum Mol Genet.; 18(6), 1099–1109. doi: 10.1093/hmg/ddp006

Tanaka, H., Iguchi, N., Toyama, Y., Kitamura, K., Takahashi, T., Kaseda, K., et al. (2004). Mice deficient in the axonemal protein Tektin-t exhibit male infertility and immotile-cilium syndrome due to impaired inner arm dynein function. Mol Cell Biol.;24(18), 7958–7964. doi: 10.1128/MCB.24.18.7958-7964.2004

Tarkar, A., Loges, N. T., Slagle, C. E., Francis, R., Dougherty, G. W., Tamayo, J. V., et al. (2013). DYX1C1 is required for axonemal dynein assembly and ciliary motility. Nat Genet.;45(9), 995–1003. doi: 10.1038/ng.2707

Teves, M. E., Zhang, Z., Costanzo, R. M., Henderson, S. C., Corwin, F. D., Zweit, J., et al. (2013). Sperm-associated antigen–17 gene is essential for motile cilia function and neonatal survival. Am J Respir Cell Mol Biol.;48(6), 765–772. doi: 10.1165/rcmb.2012-0362OC

Thoma, C.R., Frew, I.J., Hoerner, C.R., Montani, M., Moch, H., Krek, W. (2007) pVHL and GSK3beta are components of a primary cilium-maintenance signalling network. Nat Cell Biol; 9:588-595. doi: 10.1038/ncb1579

Thul, P.J., Åkesson, L., Wiking, M., Mahdessian, D., Geladaki, A., Ait Blal, H. (2017) A subcellular map of the human proteome. Science.; 356(6340). pii: eaal3321. doi: 10.1126/science.aal3321.

Tu, C.-T., Yang, T.-C., Huang, H.-Y., & Tsai, H.-J. (2012). Zebrafish arl6ip1 is required for neural crest development during Embryogenesis. PLoS ONE; 7(3), e32899. doi: 10.1371/journal.pone.0032899

Uhlén, M., Fagerberg, L., Hallström, B.M., Lindskog, C., Oksvold, P., Mardinoglu, A., et al. (2015) Tissue-based map of the human proteome. Science.; 347 (6220) : 1260419. doi: 10.1126/science.1260419. webpage:  [www.proteinatlas.org](https://www.proteinatlas.org/)

Uhlen, M., Oksvold, P., Fagerberg, L., Lundberg, E., Jonasson, K., Forsberg, M. (2010). Towards a knowledge-based Human Protein Atlas. Nat Biotechnol.; 28:1248-50. doi: 10.1038/nbt1210-1248.

van Dam, T.J., Wheway, G., Slaats, G.G., Huynen, M.A., Giles, R.H. (2013) The SYSCILIA gold standard (SCGSv1) of known ciliary components and its applications within a systems biology consortium. Cilia.; 2:7. doi:10.1186/2046-2530-2-7

VanHook, A.M. (2012) Wnt Signaling and Cilia Intertwined. Sci Signal.; 5(207):ec22. doi: 10.1126/scisignal.2002860

Vogel, P., Read, R.W., Hansen, G.M., Payne, B.J., Small, D., Sands, A.T., et al. (2012) Congenital hydrocephalus in genetically engineered mice. Vet Pathol. 49(1):166-81. doi: 10.1177/0300985811415708

Wang, H., Zou, X., Wei, Z., Wu, Y., Li, R., Zeng, R., et al. (2015) Hsp90α forms a stable complex at the cilium neck for the interaction of signalling molecules in IGF-1 receptor signalling. J Cell Sci.; 128: 100-108; doi: 10.1242/jcs.155101

Wheway, G., Schmidts, M., Mans, D. A., Szymanska, K., Nguyen, T.-M. T., Racher, H., et al. (2015). An siRNA-based functional genomics screen for the identification of regulators of ciliogenesis and ciliopathy genes Nat Cell Biol.; 17(8), 1074–1087. doi: 10.1038/ncb3201

Wilson, G.R., Wang, H.X., Egan, G.F., Robinson, P.J., Delatycki, M.B., O'Bryan, M.K. et al. (2010) Deletion of the Parkin co-regulated gene causes defects in ependymal ciliary motility and hydrocephalus in the quakingviable mutant mouse. Hum Mol Genet.;19(8):1593-602. doi: 10.1093/hmg/ddq031

Wirschell, M., Olbrich, H., Werner, C., Tritschler, D., Bower, R., Sale, W., et al. (2013). The nexin-dynein regulatory complex subunit DRC 1 is essential for motile cilia function in algae and humans. Nat Genet.;45(3), 10.1038/ng.2533. doi: 10.1038/ng.2533

Wu, M., Yang, C., Tao, B., Bu, S., & Guay-Woodford, L. M. (2013). The ciliary protein cystin forms a regulatory complex with Necdin to modulate Myc Expression. PLoS ONE.; 8(12), e83062. doi: 10.1371/journal.pone.0083062

Yang, C., Owen, H. A., & Yang, P. (2008). Dimeric heat shock protein 40 binds radial spokes for generating coupled power strokes and recovery strokes of 9 + 2 flagella. J Cell Biol.;180(2), 403–415. doi: 10.1083/jcb.200705069

Yates, B., Braschi, B., Gray, K., Seal, R., Tweedie, S., Bruford, E. (2017) Genenames.org: the HGNC and VGNC resources in 2017. Nucleic Acids Res.; 45(D1):D619-625.  doi: 10.1093/nar/gkw1033

Yen, H.J., Tayeh, M.K., Mullins, R.F., Stone, E.M., Sheffield, V.C., Slusarski, D.C. (2006) Bardet-Biedl syndrome genes are important in retrograde intracellular trafficking and Kupffer's vesicle cilia function. Hum Mol Genet.;15(5):667-77. doi: 10.1093/hmg/ddi468

Zariwala, M. A., Gee, H. Y., Kurkowiak, M., Al-Mutairi, D. A., Leigh, M. W., Hurd, T. W., et al. (2013). ZMYND10 is mutated in primary ciliary dyskinesia and interacts with LRRC6. Am J Hum Genet.; 93(2), 336–345. doi: 10.1016/j.ajhg.2013.06.007

Zhang, Q., Liu, Q., Austin, C., Drummond, I., & Pierce, E. A. (2012). Knockdown of ttc26 disrupts ciliogenesis of the photoreceptor cells and the pronephros in zebrafish. Mol Biol Cell. ;23(16), 3069–3078. doi: 10.1091/mbc.E12-01-0019
